# Phosphoproteomic Analyses Reveal Signaling Pathways That Facilitate Lytic Gammaherpesvirus Replication

**DOI:** 10.1371/journal.ppat.1003583

**Published:** 2013-09-19

**Authors:** James A. Stahl, Shweta S. Chavan, Jeffrey M. Sifford, Veronica MacLeod, Daniel E. Voth, Ricky D. Edmondson, J. Craig Forrest

**Affiliations:** 1 Dept. of Microbiology and Immunology, University of Arkansas for Medical Sciences, Little Rock, Arkansas, United States of America; 2 Myeloma Institute for Research and Therapy, University of Arkansas for Medical Sciences, Little Rock, Arkansas, United States of America; 3 UALR/UAMS Joint Program in Bioinformatics, University of Arkansas for Medical Sciences, Little Rock, Arkansas, United States of America; University of Texas Southwestern Medical Center, United States of America

## Abstract

Lytic gammaherpesvirus (GHV) replication facilitates the establishment of lifelong latent infection, which places the infected host at risk for numerous cancers. As obligate intracellular parasites, GHVs must control and usurp cellular signaling pathways in order to successfully replicate, disseminate to stable latency reservoirs in the host, and prevent immune-mediated clearance. To facilitate a systems-level understanding of phosphorylation-dependent signaling events directed by GHVs during lytic replication, we utilized label-free quantitative mass spectrometry to interrogate the lytic replication cycle of murine gammaherpesvirus-68 (MHV68). Compared to controls, MHV68 infection regulated by 2-fold or greater ca. 86% of identified phosphopeptides – a regulatory scale not previously observed in phosphoproteomic evaluations of discrete signal-inducing stimuli. Network analyses demonstrated that the infection-associated induction or repression of specific cellular proteins globally altered the flow of information through the host phosphoprotein network, yielding major changes to functional protein clusters and ontologically associated proteins. A series of orthogonal bioinformatics analyses revealed that MAPK and CDK-related signaling events were overrepresented in the infection-associated phosphoproteome and identified 155 host proteins, such as the transcription factor c-Jun, as putative downstream targets. Importantly, functional tests of bioinformatics-based predictions confirmed ERK1/2 and CDK1/2 as kinases that facilitate MHV68 replication and also demonstrated the importance of c-Jun. Finally, a transposon-mutant virus screen identified the MHV68 cyclin D ortholog as a viral protein that contributes to the prominent MAPK/CDK signature of the infection-associated phosphoproteome. Together, these analyses enhance an understanding of how GHVs reorganize and usurp intracellular signaling networks to facilitate infection and replication.

## Author Summary

Systems-level evaluations of infection-related changes to host phosphoprotein networks are not currently available for any gammaherpesvirus (GHV). Here we describe a quantitative phosphoproteomic analysis of productive GHV replication that demonstrates alterations in the phosphorylation status of more than 80% of host phosphoproteins and identifies 18 viral phosphoproteins. Systematic bioinformatics analyses reveal a predominance of MAPK and CDK signaling events within infected cells and suggest a virus-induced reorganization of signal-transduction pathways within the host phosphoprotein network. Functional experiments confirmed that CDKs and ERK MAPKs facilitate efficient viral replication and identify transcription factor c-Jun as a potential downstream target contributing to MHV68 replication. Finally, we identify the viral cyclin D ortholog as a major pathogen-encoded factor contributing to the MAPK/CDK signature of the infected cell phosphoproteome. These data provide new insight into both viral and host factors that regulate phosphorylation-dependent signaling during lytic GHV replication and offer a new resource for better defining host-pathogen interactions in general.

## Introduction

Post-translational modification of proteins by phosphorylation and dephosphorylation regulates numerous functional properties, including activation status [1], stability [2], protein-protein interactions [3], and subcellular localization [4]. Such signals regulate the majority of cellular processes ranging from cell-cycle progression [5], [6] to terminal differentiation of specific cell types [7] to activation of intracellular signals that trigger both local and organismal antimicrobial responses [8]. Following infection of host cells, viruses and intracellular bacteria manipulate cellular signaling to facilitate replication. Pathogen-directed signaling may mobilize enzymatic pathways to provide nutrients or energy necessary for the large increase in macromolecular biosynthesis [9] or reorganize host components to direct packaging, envelopment, or egress [10]. In defense, host-cell sensing of microbial infection may trigger signaling cascades aimed at hindering pathogen replication and alerting neighboring cells to the present danger [8]. Pathogens also may encode factors to deregulate anti-microbial signaling pathways in order to prevent detection or elimination by host immune responses [11].

Recent innovations coupling affinity-based phosphopeptide enrichment with high-resolution mass spectrometry followed by systematic bioinformatics analyses have enabled systems-level evaluations of phosphorylation-dependent signaling cascades in cells or tissues responding to discrete stimuli, such as epidermal growth factor receptor stimulation or DNA damage responses (DDR) [12], [13]. Such analyses revealed that >90% of detectable phosphorylation sites on cellular phosphoproteins were not previously identified [13] and that critical regulatory phospho-motifs and phosphorylated effector proteins remain to be identified, even for extensively studied signaling cascades [12], [13]. Currently, systems-level phosphoproteomic analyses to define infection-associated alterations in protein phosphorylation status during viral infection are lacking. Thus, while hypothesis-driven and intuition-based studies have identified many phosphorylation-dependent signaling events that regulate viral replication and host responses to infection, it is likely that the vast majority of infection-associated changes in host protein phosphorylation status are not yet known. This highlights a critical gap in our current understanding of virus-host interactions. Importantly, the identification of unappreciated signaling pathways and/or effector proteins usurped or inhibited by pathogens in infectious disease states may reveal new targets for pharmacologic intervention.

Gammaherpesviruses (GHVs) are members of the *Herpesviridae* family of large double-strand DNA viruses [14]. GHVs include the human pathogens Epstein-Barr virus (EBV) and Kaposi sarcoma-associated herpesvirus (KSHV or HHV-8); non-human primate viruses herpesvirus Saimiri (HVS), rhesus rhadinovirus (RRV), and rhesus lymphocryptovirus (rhLCV); and rodent pathogens wood mouse herpesvirus (WMHV), rodent herpesvirus Peru (RHVP), and murine gammaherpesvirus-68 (γHV68 or MHV68). Like all herpesviruses, GHVs exhibit two distinct phases of their infectious cycles. The productive replication phase (also termed lytic replication) is characterized by robust viral gene expression leading to viral DNA replication and the production of infectious progeny virions. In contrast, latent infections are characterized by restricted viral gene expression and indefinite maintenance of the viral genome as episomal DNA. GHVs characteristically establish lifelong latent infections of lymphocytes, thereby placing the host at risk for lymphoid and other cancers, especially in settings of immunocompromise such as HIV infection or immunosuppression for organ transplants [15], [16], [17].

In contrast to EBV and KSHV, MHV68 – which provides a tractable small animal model for evaluating GHV pathogenesis – undergoes robust productive replication in cultured cells. Further, given the ease of generating MHV68 mutants and the capacity to perform controlled experimental infections of various wild-type (WT), knockout, and transgenic mice, MHV68 offers an attractive system for understanding the virus-host dynamic during productive viral replication [18], [19], [20]. Akin to what is hypothesized to occur during primary EBV infection of humans, acute MHV68 infection of mucosal epithelia following intranasal inoculation is necessary for viral dissemination and latency establishment in distal reservoirs [21], [22], [23]. While the importance of DDR, mitogen-activated protein kinase (MAPK), and inhibitor of kappa-B kinase (IKK) signaling in MHV68 replication recently was demonstrated [24], [25], [26], [27], an understanding of how the kinases involved in these responses influence the overall phosphoprotein milieu within the host cell is not known. The breadth of host or viral proteins targeted by specific viral signaling proteins with critical roles in pathogenesis, such as the conserved herpesvirus protein kinase (CHPK) ORF36 [28] or the viral cyclin D ortholog, encoded by gamma-2-herpesviruses such as KSHV [29] and MHV68 [30], also has not been globally evaluated. Hence, our understanding of GHV-regulated signaling events – as well as host cell responses to infection – in facilitating productive viral replication and ultimately pathogenesis is incomplete. To this point, a better appreciation of phosphorylation-dependent signaling during GHV replication may illuminate novel targets for the treatment of infectious mononucleosis, the acute phase malady of primary EBV infection [31], or Kaposi sarcoma, a KSHV-related cancer for which lytic viral replication is thought to be a driver of disease [32], [33].

In this study, we use a comparative, quantitative phosphoproteomic analysis to define phosphorylation-related signaling events regulated during productive MHV68 infection. We identified more than 400 host phosphoproteins that are induced or repressed in infected cells, as well as 17 viral phosphoproteins. Quantitative analyses indicated that a vast majority of definable phosphopeptides was regulated during GHV infection. Unbiased bioinformatics analyses and complementary biochemical approaches predicted the importance of extracellular-signal related and cyclin-dependent kinases (ERK and CDK, respectively) in facilitating viral replication, and pharmacologic inhibition and shRNA knockdown experiments confirmed the predictions. Finally, we identified the MHV68 cyclin ortholog as a key contributor to many of the virus-associated signaling changes observed. Together, these data and analyses provide a novel systems-level resource to better inform and enable studies of pathogen-host interactions.

## Results

### Comparative phosphoproteomic analysis of control and infected cells

**Figure 1:**
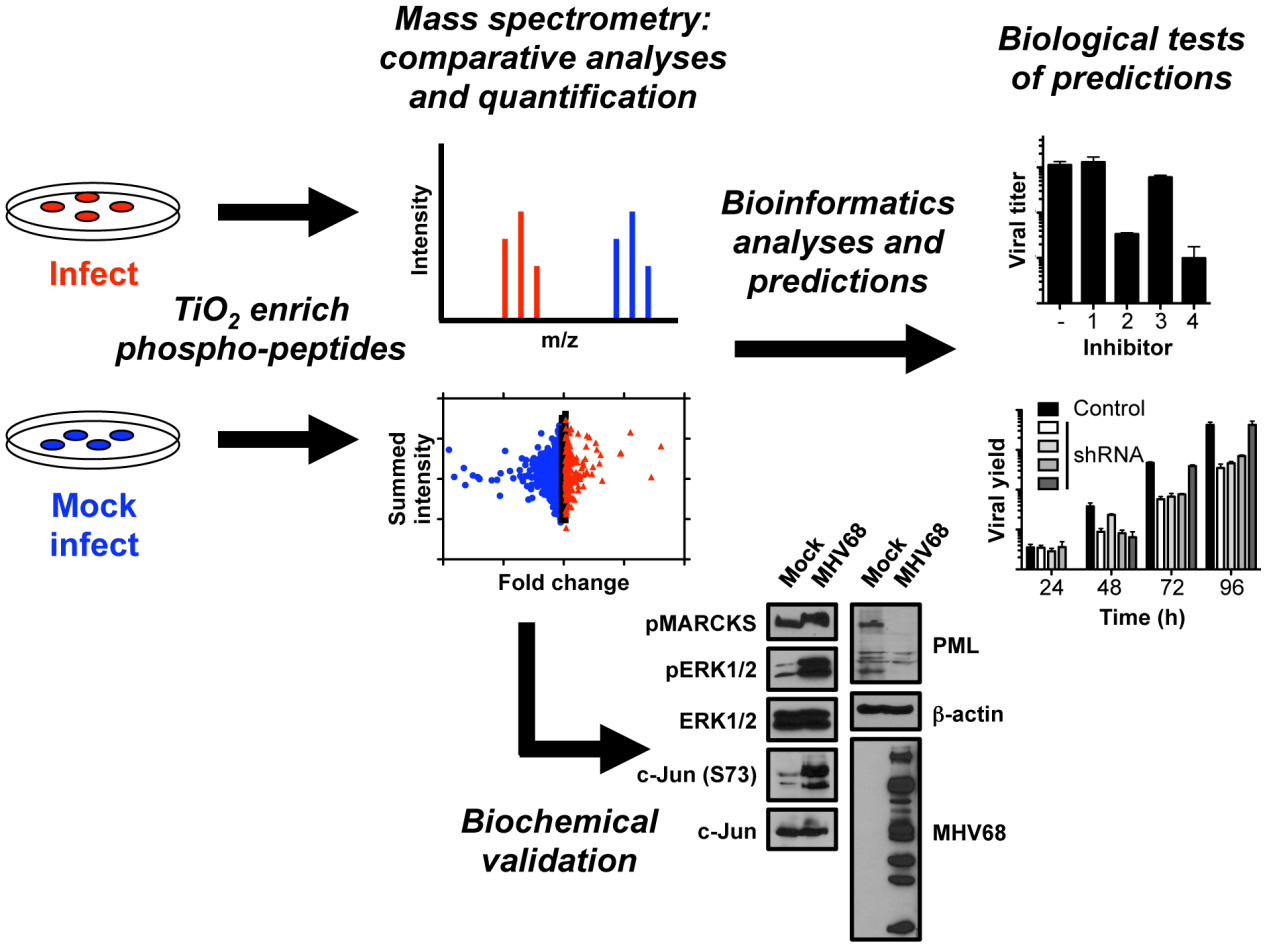
Overview of integrated approach combining phosphoproteomics, bioinformatics, and biological tests to define signaling events that regulate MHV68 lytic replication.

**Figure 2:**
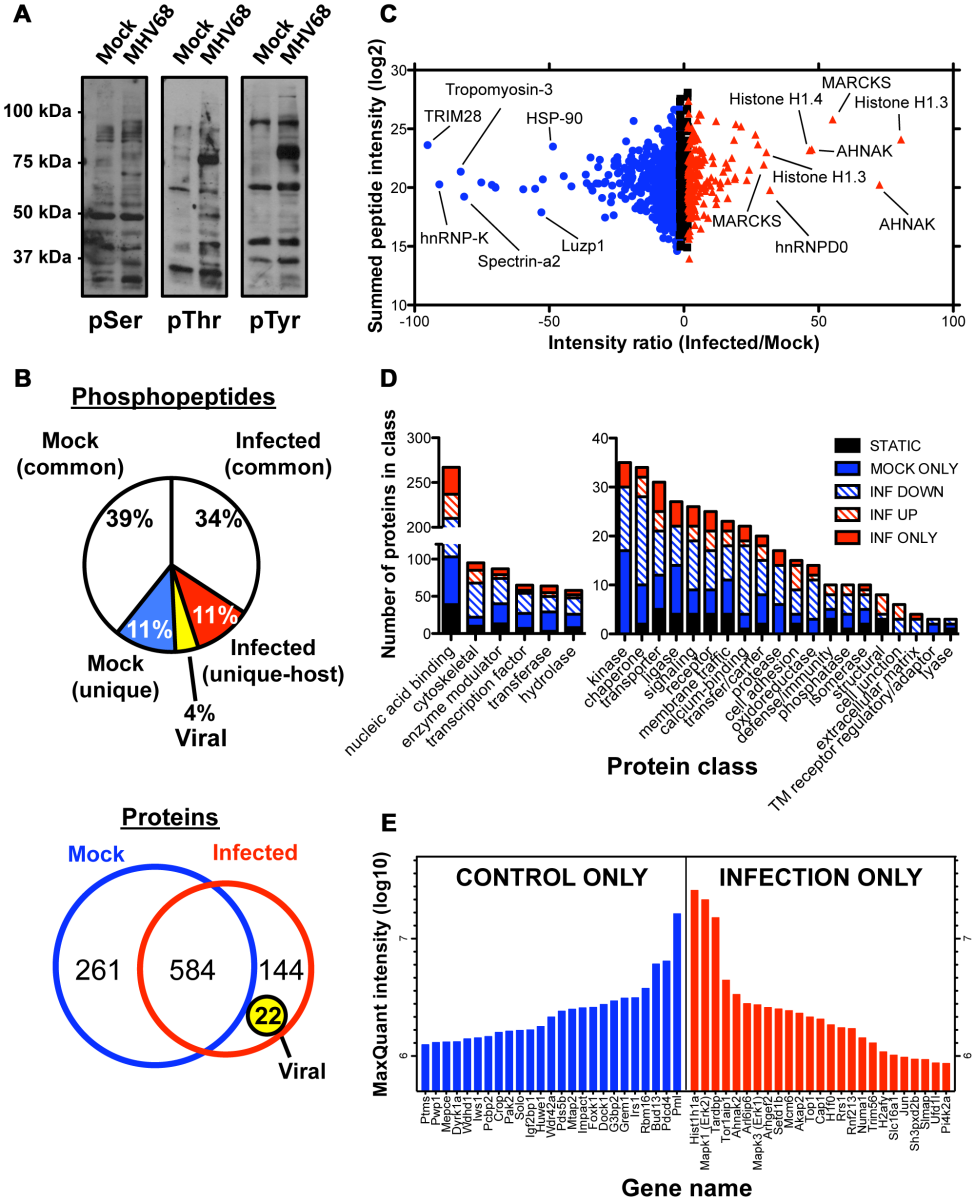
Label-free, quantitative phosphoproteomic analyses identify phosphoproteins induced and repressed in MHV68 infection. (A) Serum-starved 3T3 fibroblasts were mock-infected or infected at MOI = 5 PFU/cell, and cells were harvested 18 h post-infection, the timepoint for which tryptic peptides were enriched by TiO_2_ IMAC and identified by high resolution mass spectrometry. Proteins were resolved by SDS-PAGE, and immunoblot analyses were performed using antibodies to p-Ser, p-Thr, or p-Tyr to biochemically demonstrate phosphoproteomic changes induced by MHV68 infection. (B) Pie chart depicts percentages of phosphopeptides that are unique to or shared between control and infected systems. Venn diagram depicts the relative numbers of proteins and overlap of the control and infected proteomic data sets. (C) Scatter plot demonstrating changes in relative abundance for specific phosphopeptides following MHV68 infection. Black squares represent unchanged peptide abundance, blue circles indicate peptides exhibiting >1.5-fold reduction in abundance during infection, and red triangles indicate peptides exhibiting >1.5-fold increased abundance during infection. (D) Phosphoproteins were analyzed using the PANTHER database to classify each protein by “Protein Class” gene ontology (GO). Ontologically-associated proteins are labeled according to their relative abundance in global phosphoproteomic data sets to visualize how infection-related phosphorylation events target each represented GO class. (E) The identities and MaxQuant-defined intensities of the 50 highest scoring phosphopeptides that were either lost (Control ONLY) or induced (Infection ONLY) during MHV68 infection.

To gain a more complete understanding of GHV-induced changes in host cell signaling networks, we devised an experimental approach integrating high-resolution mass spectrometry, predictions based on orthogonal bioinformatics analyses, and hypothesis-driven functional tests (Fig. 1). We first performed time-course immunoblot analyses using phospho-residue-specific antibodies to determine timepoints at which MHV68 elicited robust changes in protein phosphorylation patterns during productive infection of mouse fibroblasts. These experiments revealed the greatest qualitative differences in phosphoprotein detection between control and infected samples at 18 h post-infection (Fig. 2A and S1). At this timepoint, phosphopeptides were selectively enriched from mock-infected and infected cells using titanium dioxide (TiO_2_) chromatography. Three separate enrichments were performed for each of two independent experiments, and peptides were sequenced by HPLC-coupled high-resolution MS/MS analysis using collision-induced dissociation on an LTQ Orbitrap hybrid mass spectrometer. The analysis identified 13,801 peptides, ca. 80% of which contained detectable phosphorylated residues. As only 30% of the cellular proteome is estimated to be phosphorylated [34], this indicates the enrichment procedure was robust. The processed and sorted data sets are available as Table S1. Despite identifying peptides from 14% fewer distinct proteins in infected samples, the absolute numbers of phosphopeptides identified for control and infected samples was essentially equivalent (5632 vs. 5472, respectively), which indicates that the enrichment and analysis procedures did not introduce bias. Notably, approximately 15% of phosphopeptides identified from infected cells are specific to the infected state (i.e., only detected in infected analytes), 4% of which derive from viral proteins, whereas 11% of control phosphopeptides were not detected in infected cells (Fig. 2B). Extended to the protein level, we identified a total of 989 individual proteins representing 845 proteins from control cells and 728 proteins from infected cells (Fig. 2B). 584 of the identified proteins were present in both control and infected samples, while 261 or 144 proteins were unique to either control or infected cells, respectively (Fig. 2B). 22 of the 144 infection-specific proteins (ca. 15%) were derived from MHV68.

For quantitative analyses, the relative intensity (a quantitative measure of protein abundance) for each identified peptide was determined using MaxQuant [13]. Of the 2428 unique peptides identified, 86% exhibited greater than 2-fold change in peptide intensity between control and infected cells. A dot plot of summed peptide intensity vs. intensity ratio demonstrates the extent to which common phosphopeptides – those identified in both control and infected samples – are up or down-regulated during MHV68 infection (Fig. 2C). By comparison, recent studies to define phosphoproteomic changes induced by discrete stimuli, such as growth factor stimulation or DNA damage, found that less than 15% of phosphoproteins were regulated (enhanced or decreased phosphorylation) in response to stimulus [12], [13]. Similarly, Hela cell challenge with *Salmonella* also elicited a more modest change in host-cell phosphoprotein status than MHV68 infection, with ca. 24% of phosphopeptides being regulated [35]. The breadth of differential phospho-protein regulation induced by MHV68 is further highlighted by gene ontology (GO) analyses, which revealed that infection-associated changes in protein phosphorylation status were evident in essentially all ontologically-grouped protein classes, rather than effecting specific types of proteins (Fig. 2D). Interestingly, all MS-identified proteins annotated to the “kinase” protein class exhibited phosphorylation status changes during MHV68 infection (Fig. 2D). This also was true for all proteins annotated to “protease” and “oxidoreductase” protein classes (Fig. 2D). Thus, MHV68 infection effects dramatic changes in the host-cell phosphoproteome on a scale not previously observed in comparable phosphoproteomic analyses.

Several host proteins are notable among those specifically induced in or lost from infected cells. The promyelocytic leukemia protein (PML) is the single most abundant phosphoprotein detected in control cells, yet absent from infected cell analytes (Fig. 2E). This finding is consistent with PML being targeted for degradation by MHV68 tegument protein, ORF75C [36], [37]. Infected samples, on the other hand, displayed highest levels of phosphorylated linker histone H1 variant 1, MAPK1/ERK2, and TAR DNA binding protein (TARDBP), a protein originally identified as a host factor that enhances HIV transcription [24]. MAPK3/ERK1 and MAPK substrate c-Jun also were exclusive to infected cells. Although roles for ERK1/2 in MHV68 replication have not been described, ERK1/2 were previously implicated in facilitating KSHV replication [38], [39], [40]. Further, we recently demonstrated c-Jun phosphorylation and related AP-1 transcription factor activation during MHV68 replication [24]. Thus, several MS-defined phosphoproteins identified in our study are consistent with previously published findings, thereby providing confidence in the phosphoproteomic data sets obtained.

We also identified 18 viral phosphoproteins and non-phosphorylated peptides derived from 5 viral proteins (Table 1). Of the viral phosphoproteins identified, to our knowledge only tyrosine phosphorylation of ORF21/thymidine kinase (TK) was previously reported for MHV68 [41]. In addition to defining the specific phospho-tyrosine residues previously reported, we also identified numerous phosphorylation events on serine and threonine residues within ORF21/TK (Table 1). Six of the MHV68 phosphoproteins we identified [ORF8/gB, ORF21/TK, ORF25/major capsid protein (MCP), ORF27/gp48, ORF39/gM, and ORF45] correspond to homologous phosphoproteins present in purified EBV virions [42]. Phosphorylation of ORF21/TK [41], ORF45 [43] and viral DNA polymerase processivity factor ORF59 [44] also were demonstrated during KSHV infection. Interestingly, phosphorylation of pUL44, the HCMV homolog of ORF59 [45], [46], [47], [48], and glycoprotein B (gB) phosphorylation, during both HCMV and HSV1 infection [49], [50] suggest that some of these phosphorylation events may be functionally conserved among all herpesvirus subclasses. Also identified were phosphorylated peptides for ORF36, a conserved herpesvirus protein kinase which by analogy to HCMV and HSV should be capable of autophosphorylation [28]. The identification of discretely phosphorylated residues in viral proteins, especially those conserved for other herpesviruses, provides a resource for future functional studies to define roles for specific phospho-motifs and related kinases in the processes of infection, persistence, and pathogenesis.

**Table 1:** Viral phosphopeptides identified through phosphoproteomic analyses.

Protein	Possible Function	Phosphopeptide sequence	# of peptides	Phospho Motifs
ORF6	Single-strand DNA binding protein	RIAT*FEDIDL	6	CAMK2;PKA/AKT;FHA1 Rad53p
ORF8	Glycoprotein B	GYSQLPLEDESTS*L	5	
		QAPPPYS*ASPPAIDKEEIKR	1	
ORF10		AIT*DGGES*PMEWQTL	5	CK2;CAMK2
ORF11		AESHPWS*GRPSSS*PR	10	CK1;GSK3;PKA; CAMK2;CDK2;CDK1; Polo box
ORF17	Capsid Protein	VQQLFCEELS*K	3	
ORF21	Thymidine Kinase	VSS*VGENYDIPR	6	PKA;CK1;CK2;CAMK2; PKD;CHK1;GSK3; CDK2;ERK/MAPK; CDK1;PLK1;FHA1 Rad53p
		MPGT*PSR	5	
		HPEAGGNKPDFLPVDKPLPS*VPQHVPQGYEEMAGS*PPPER	7	
		IITRRPS*T*GEVER	4	
		RPS*T*GEVER	16	
		NNPGFQPDS*DS*DEHVY*AEPEEVYDNPFDAMR	26	
		QEAVDS*DDDATYAT*PSFDR	16	
		RPNPPVIIFS*DS*ET*ESDAEAVGER	44	
		RPPEQSDS*ES*DY*EDIGAYGQPAR	23	
		HPEAGGNKPDFLPVDKPLPSVPQHVPQGY*EEMAGSPPPER	1	
		RPPEQSDSESDY*EDIGAYGQPAR	2	
		VSSVGENY*DIPR	1	
		NNPGFQPDSDSDEHVY*AEPEEVYDNPFDAMR	3	
ORF25	Major Capsid Protein	KIMAELIT*MEQT*LLKICGHEK	1	PLK1
ORF27	Glycoprotein 48	NS*SGEVVLNGPYHCSENSR	1	PKA;CK2;CAMK2;PKD; CHK1/2;CHK1
ORF36	Kinase	VSTMAS*DDTLCIK	9	CK1;FHA1 Rad53p
		FT*PGDTLGEGGFGR	1	
ORF39	Glycoprotein M	AKY*TDLET*ES*EDEL	20	CK2
ORF45		ETQSDSSSDSS*GNSHK	1	CK1;GSK3;CK2
ORF52	Tegument Protein	RES*IIVSSSR	4	PKA;GSK3;AURORA; AURORA-A;CK1;CK2
		VKSSGAVS*S*DDSILTAAKR	15	
		VDVSMEETTVGASGGIGPS*S*QTET*KK	19	
ORF59	DNA replication	LSVALLNVDSS*TGR	3	CK2;CAMK2;AURORA; CK1;GSK3;PKA; FHAKAPP;NEK6;FHA1 Rad53p
		DRGAS*KS*EDADGS*PPVPDHPKES*LT*SDSDSSVPGPR	76	
		VLKEPVVT*GAS*KDR	33	
		ES*LT*S*DS*DSSVPGPR	15	
		S*TDLS*S*LT*HT*PS*DRFS*SDLR	66	
ORF64	Tegument Protein	QIVS*PVITILPGTK	1	
ORF65	M9	VIPDPSS*LTDKDDQNLGESTK	1	
ORF68	Glycoprotein 67	GAQS*PDPPTGCPVK	3	
ORF69	Nuclear Egress	MRS*T*GS*APSCSGESAR	3	CK1;PKA;FHA KAPP
		STGS*APS*CSGESAR	2	
ORF75B	Tegument Protein	YFLT*SPVR	1	GSK3;CDK2;CDK1; 14-3-3 binding

### Biochemical validation of host and viral phosphoproteins regulated during MHV68 infection

**Figure 3:**
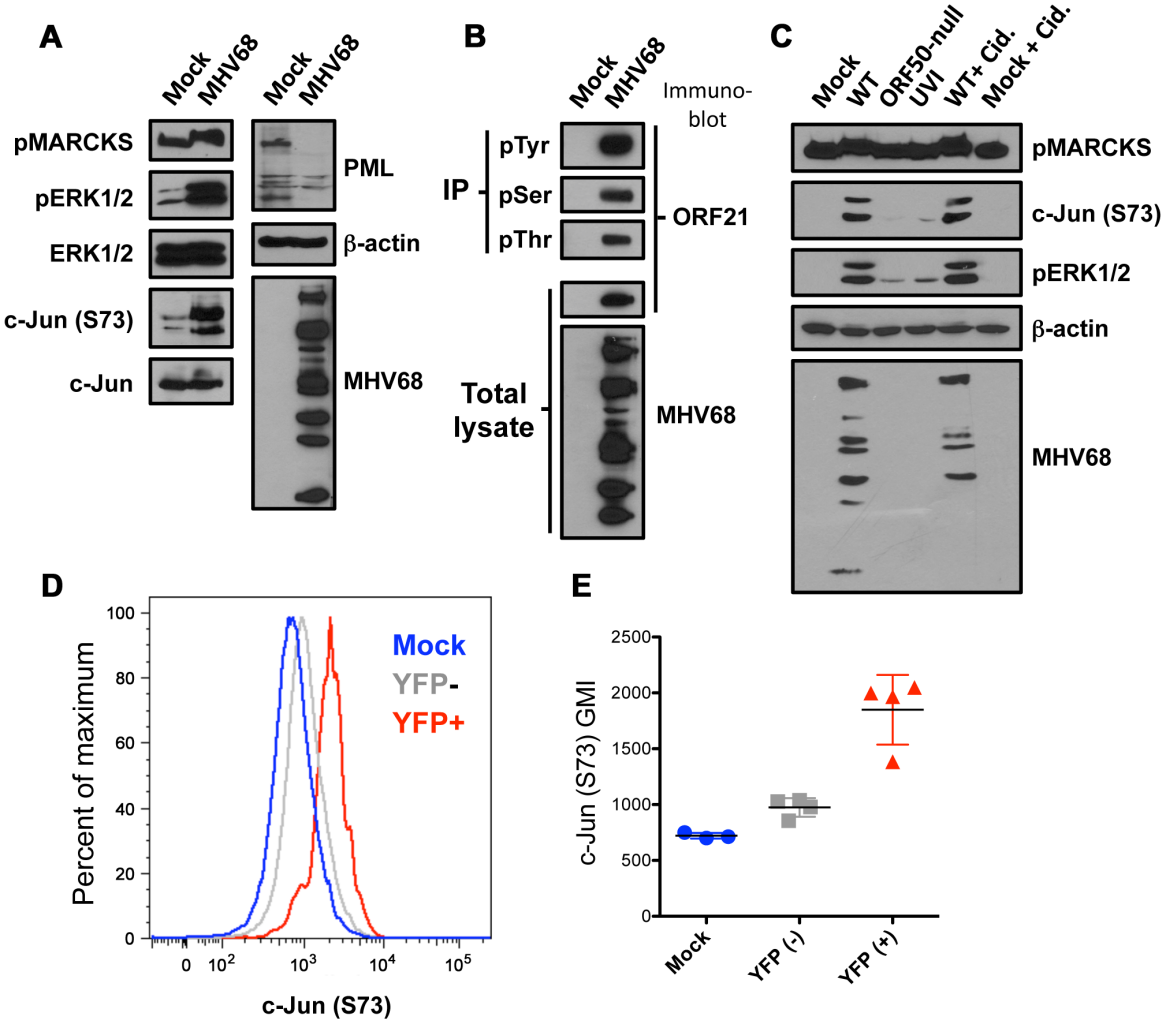
Biochemical validation of mass-spectrometry identified infection-related phosphorylation events. (A) 3T3 fibroblasts were mock-infected or infected with MHV68 at MOI = 5 PFU/cell. Cells were harvested 18 h post-infection, proteins were resolved by SDS-PAGE, and immunoblot analyses were performed using antibodies to detect the indicated phosphorylated or total proteins. (B) 3T3 fibroblasts were mock-infected or infected with MHV68 at MOI = 5 PFU/cell. Cells were harvested 18 h post-infection, and phosphoproteins were immunoprecipitated using antibodies directed against p-Tyr, p-Ser, or p-Thr. Whole cell lysates (1/40^th^) or immunoprecipitates were resolved by SDS-PAGE. Immunoblot analyses were performed to detect ORF21 protein or MHV68 lytic antigens. (C) Cells were mock infected in the presence or absence of the antiviral drug cidofovir (Cid.), or infected with WT MHV68, UV-inactivated (UVI) MHV68, ORF50-null MHV68, or WT MHV68 in the presence of cidofovir. Cells were harvested, and immunoblot analyses were performed as in A. (D and E) Mice were intraperitoneally mock-inoculated or inoculated with 10^6^ PFU of H2B-YFP-expressing MHV68. Animals were sacrificed 4 days post-infection, and bulk splenocytes were isolated and processed for flow cytometry to detect H2B-YFP and S73-phosphorylated c-Jun. A representative histogram (D) depicts phospho-S73 c-Jun detection levels in mock-infected (blue), H2B-YFP− cells from infected animals (gray), and MHV68 infected H2B-YFP+ cells (red). The H2B-YFP gating strategy and a comparison to productively-infected fibroblasts is shown in Fig. S3. Data graphed in (E) represent compiled geometric mean fluorescence intensities (GMI) from 3 mock-infected or 4 infected mice.

We next performed a series of comparative immunoblot analyses to validate the MS data. In agreement with MS determinations, ERK1/2 was potently phosphorylated following MHV68 infection of murine fibroblasts, while total ERK1/2 levels remained unchanged (Fig. 3A). Infection-associated c-Jun phosphorylation on Ser73 also was readily detected. As for differentially regulated phosphoproteins (i.e., those present at differing abundance in both control and infected MS data sets), quantitative MS analyses indicated that MHV68 infection enhanced phosphorylation of the myristoylated alanine-rich C-kinase substrate (MARCKS), a classic protein kinase C (PKC) targeted scaffold protein (highlighted in Fig. 2C). By immunoblot, infection-related enhancement of MARCKS phosphorylation manifested as a retarding of MARCKS mobility during SDS-PAGE (Fig. 3A). In contrast to proteins for which phosphorylation was enhanced during infection, PML protein only was detected in control cell lysates (Fig. 3A), which is consistent with the presence of PML phosphopeptides in control, but not infected, MS analyses. As stated above, this finding is consistent with PML protein being targeted for degradation by the MHV68 tegument protein, ORF75C [36], [37].

To validate viral phosphoproteins, we evaluated whether ORF21/TK and ORF59, the two most abundant viral phosphoproteins identified (Table 1), were phosphorylated during MHV68 infection. For ORF21, phosphoproteins were captured by immunoprecipitation with p-Thr, p-Ser, and p-Tyr-specific antibodies. Immunoblot analyses readily detected ORF21 in phospho-specific immunoprecipitates (Fig. 3B). Interestingly, ORF21 mobility in SDS-PAGE corresponded to the abundant ca. 80 kDa phosphoprotein detected in infected cell lysates (see Fig. 2A). Likewise, p-Thr and p-Ser-specific antibodies also recognized immunoprecipitated ORF59 (Fig. S2). These data provide complementary biochemical evidence that supports the identification of ORF21 and ORF59 as phosphorylated viral proteins by MS. Taken together, the results of these experiments biochemically validate the presence or absence of several viral and host phosphoproteins identified through global phosphoproteomics analyses. Thus, these data provide additional confidence in the robustness of the MS data sets.

Because our MS analyses focused on timepoints associated with robust infection-induced alterations in total phosphoprotein profiles, we next sought to correlate infection-associated phosphoproteomic changes with specific stages in the viral replication cycle. Cells were mock-infected or infected with WT MHV68, UV-inactivated (UVI) MHV68, ORF50-null (50.Stop) MHV68, or WT MHV68 in the presence of cidofovir, a nucleoside analog that blocks viral DNA replication and hinders progression into the late phase of the viral replication cycle [51]. Due to disruption of the gene encoding the viral transactivator protein, RTA, ORF50-null MHV68 arrests at the immediate-early (IE) gene expression stage [52]. Consistent with time-course analyses suggesting that the majority of infection-associated phosphoproteomic changes occur during early-to-late stages of infection (Fig. S1), ERK1/2 phosphorylation, c-Jun phosphorylation, and the retardation of MARCKS mobility only were evident in cells infected with WT MHV68 or WT MHV68 in the presence of cidofovir (Fig. 3C). The findings that UVI and ORF50-null MHV68 did not elicit changes in the phosphorylation status of the proteins tested indicate that active viral gene expression and progression through the replication cycle, not simple internalization of the virus or presumed initiation of IE transcription, respectively, are important for infection-associated induction of host phosphoproteins. Additionally, while cidofovir reduced viral protein production as evidenced by immunoblot analyses with MHV68 antiserum (Fig. 3C) and ca. 20-fold reduction in viral titers (not shown), cidofovir did not inhibit ERK1/2, c-Jun, and MARCKS phosphorylation (Fig. 3C). These data suggest that viral DNA replication, and most likely late viral gene expression, do not play major roles in the infection-related signaling events evaluated. These data agree with a previous study in which JNK1/2 and c-Jun phosphorylation occurred during MHV68 infection despite inhibition of viral DNA synthesis with phosphono-acetic acid [24]. Further, while virus-related signaling undoubtedly occurs coordinate to other steps in the infection process, results of these experiments support the notion that our phosphoproteomic analyses offer an accurate representation of signaling events corresponding to the early-to-late phases of the viral replication cycle.

Finally, in an effort to link cell culture observations to the in vivo setting, we evaluated c-Jun phosphorylation in infected cells during acute MHV68 replication. Mice were intraperitoneally (IP) inoculated with recombinant MHV68 expressing a histone H2B-YFP fusion protein as a fluorescent marker to enable detection of infected cells by flow cytometry [53]. Four days after IP inoculation with MHV68, a timepoint at which robust productive viral replication is occurring in the spleen [54], splenocytes were harvested and processed for flow cytometry to detect H2B-YFP and c-Jun phosphorylated on Ser73. The H2B-YFP gating strategy and an analogous proof-of-principle experiment in productively infected fibroblasts are provided in Figure S3. Compared to splenocytes from mock-infected animals and H2B-YFP-negative cells from infected animals, H2B-YFP-positive cells exhibited a significant increase in the detection of phosphorylated c-Jun, with an approximate 2-fold increase in mean fluorescence intensity (Figs. 3D and 3E). This approximated the induction of c-Jun phosphorylation observed in productively-infected fibroblasts in culture (Fig. S3). The finding that H2B-YFP-negative cells from infected animals exhibit phospho-c-Jun signals approximating those of mock-infected animals is important, because it demonstrates that c-Jun phosphorylation during acute MHV68 infection occurs specifically in infected cells, rather than through a non-specific bystander process such as immune activation [55], [56]. These findings provide an important experimental link suggesting that signaling parallels exist between productive replication in culture and during MHV68 pathogenesis in vivo.

### Infection reorganizes the host phosphoprotein network

Having validated the initial MS data sets, we next performed a battery of bioinformatics analyses to determine the functional consequences of phosphoproteomic changes during MHV68 infection and potential signaling pathways involved. Given the extent to which infection altered the phosphorylation status of host proteins, we first sought to determine whether infection elicited a rearrangement of the global cellular phosphoprotein network. A central tenet of systems biology analyses is the assertion that most if not all components of a complex network exert some influence over other components of the system [57], [58]. As such, network analyses predict that changes in the “interactions” (i.e., functional association as defined by many disparate properties) between specific molecules, especially those positioned downstream of multiple stimuli or between functional protein modules, can have dramatic influences on the propagation of signals through, and ultimately the response of, a particular system [57], [58]. While numerous linear analyses of signaling pathways that regulate GHV replication have been performed, how infection by GHVs modulates the host phosphoprotein network is not known. We therefore performed comparative network analyses to provisionally identify critical host molecules and protein complexes that were manipulated and reorganized during MHV68 infection.

We first performed STRING (Search Tool for the Analysis of Interacting Genes/Proteins) analyses [59] to establish global phosphoprotein interaction networks for all proteins detected in either control or infected cells (Figs. S4 and S5, respectively). The STRING algorithm links genes or proteins into networks based on published functional or informatics-predicted interactions [59]. Phosphoprotein networks were then processed in Cytoscape [60], and interconnected proteins (referred to as nodes) were color coded based on MaxQuant intensities to denote detection and/or relative abundance. Node size correlates to betweenness centrality (BC), a measure of a protein's capacity to connect disparate protein modules in the network [61], thus allowing a visualization of nodes that likely have a strong influence on signal propagation through the networks. The width of connecting arrows between nodes (known as edges) represents the STRING-defined confidence value of the predicted interaction. Disconnected nodes, those that did not “interact” with any other proteins evaluated (see Fig. S4 and S5), are not shown.

**Figure 4:**
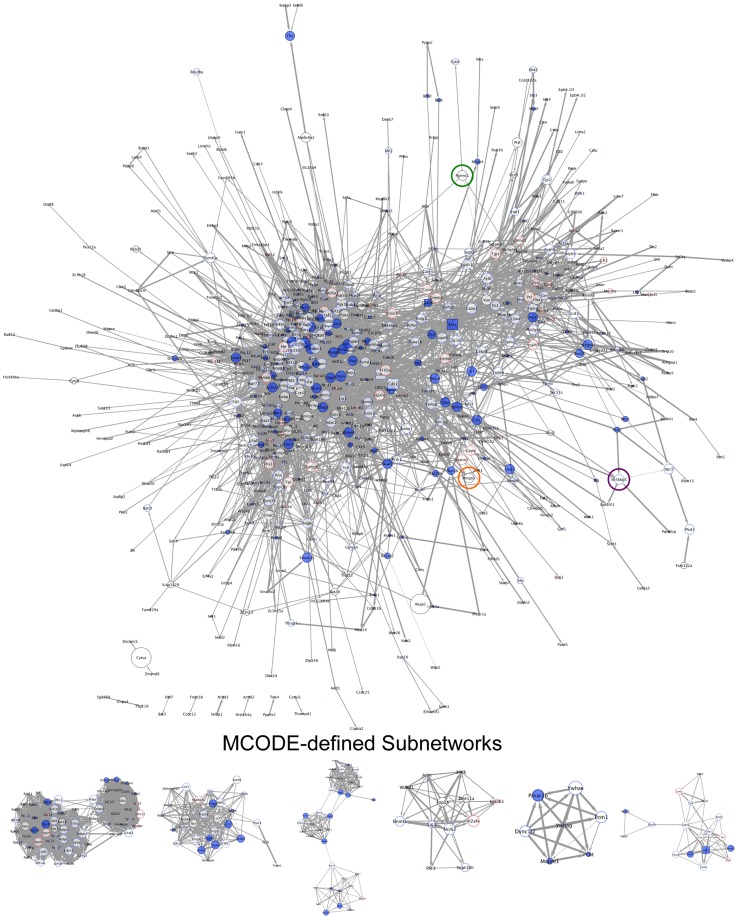
Global phosphoprotein network of uninfected cells. Interaction-network analyses were performed for all proteins identified in control data sets using STRING to define a global phosphoprotein network. Standard STRING-defined confidence values of 0.40 were used as cut-offs for putative interactions. All connected nodes were imported into Cytoscape, and specific identifiers were assigned to each node according to MaxQuant values. Solid red (see Fig. 5) denotes detection only in infected cells. Solid blue denotes detection only in control cells. Red borders denote increased abundance during infection. Blue borders denote reduced abundance during infection. Black borders indicate no infection-related change in protein intensity. Gene names for each protein are shown. Kinases are depicted as square nodes. Node size corresponds to betweenness centrality, a measure of a particular node's capacity to connect disparate protein clusters within the network. Edge weight corresponds to STRING-defined confidence values, where a thicker line indicates a higher confidence prediction. Nodes highlighted with green, orange, or purple circles represent common nodes in both control and infected networks (see Fig. 5) and are provided to facilitate orientation. Subnetworks represent the 6 highest scoring functional protein clusters identified within each global network using MCODE.

**Figure 5:**
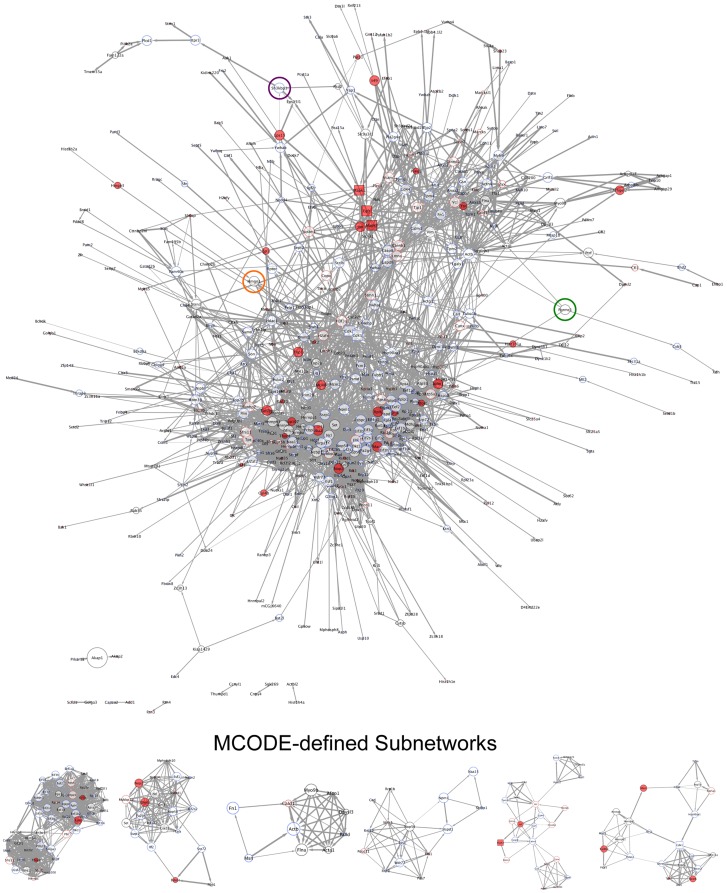
Global phosphoprotein network of MHV68-infected cells. Interaction-network analyses were performed for all proteins identified in infected data sets using STRING to define a global phosphoprotein network. Standard STRING-defined confidence values of 0.40 were used as cut-offs for putative interactions. All connected nodes were imported into Cytoscape, and specific identifiers were assigned to each node according to MaxQuant values. Solid red denotes detection only in infected cells. Solid blue (see Fig. 4) denotes detection only in control cells. Red borders denote increased abundance during infection. Blue borders denote reduced abundance during infection. Black borders indicate no infection-related change in protein intensity. Gene names for each protein are shown. Kinases are depicted as square nodes. Node size corresponds to betweenness centrality, a measure of a particular node's capacity to connect disparate protein clusters within the network. Edge weight corresponds to STRING-defined confidence values, where a thicker line indicates a higher confidence prediction. Nodes highlighted with green, orange, or purple circles represent common nodes in both control (see Fig. 4) and infected networks and are provided to facilitate orientation. Subnetworks represent the 6 highest scoring functional protein clusters identified within each global network using MCODE.

Upon gross inspection, it was immediately evident that control and infected protein networks were topologically unique (compare Figs. 4 and 5, note the repositioning of highlighted common nodes during infection relative to control). Interestingly, several proteins specifically induced (c-Jun, MAPK1/ERK2, MAPK3/ERK1, and others) or lost (AKT1, PCNA, Smad2, and others) from the infected phosphoproteome have high BC values indicated by relatively large node size (Figs. 4 and 5). These findings suggest that the induction or repression of specific proteins during infection alters the flow of information from protein to protein within the cell [57], [58]. MCODE analyses to identify functional protein clusters within the networks [62] identified either unique clusters or clusters in which the composition of proteins represented varied according to infection (Figs. 4 and 5). Indeed, none of the highest scoring clusters were identical, which further indicates reorganization of functional protein modules in the network during MHV68 lytic replication. Finally, GO analyses utilizing the Cytoscape plugin BiNGO [63] indicate that induction or repression of specific proteins during MHV68 infection alters many functionally grouped biological processes represented in the phosphoprotein network (Table S2). Of note, GO IDs associated with chromatin organization, RNA production and localization, and negative regulation of cell death were unique or overrepresented during MHV68 infection, whereas biological processes associated with hormone-related signaling pathways, ubiquitylation, and signal transduction were absent in comparison to control networks (Table S2). These findings suggest that infection-directed changes in the phosphorylation status of specific proteins redirects the flow of information through the cellular phosphoprotein network to effect broad functional changes to specific biological processes.

### Bioinformatic predictions of functional protein modules influenced by MHV68 infection

**Figure 6:**
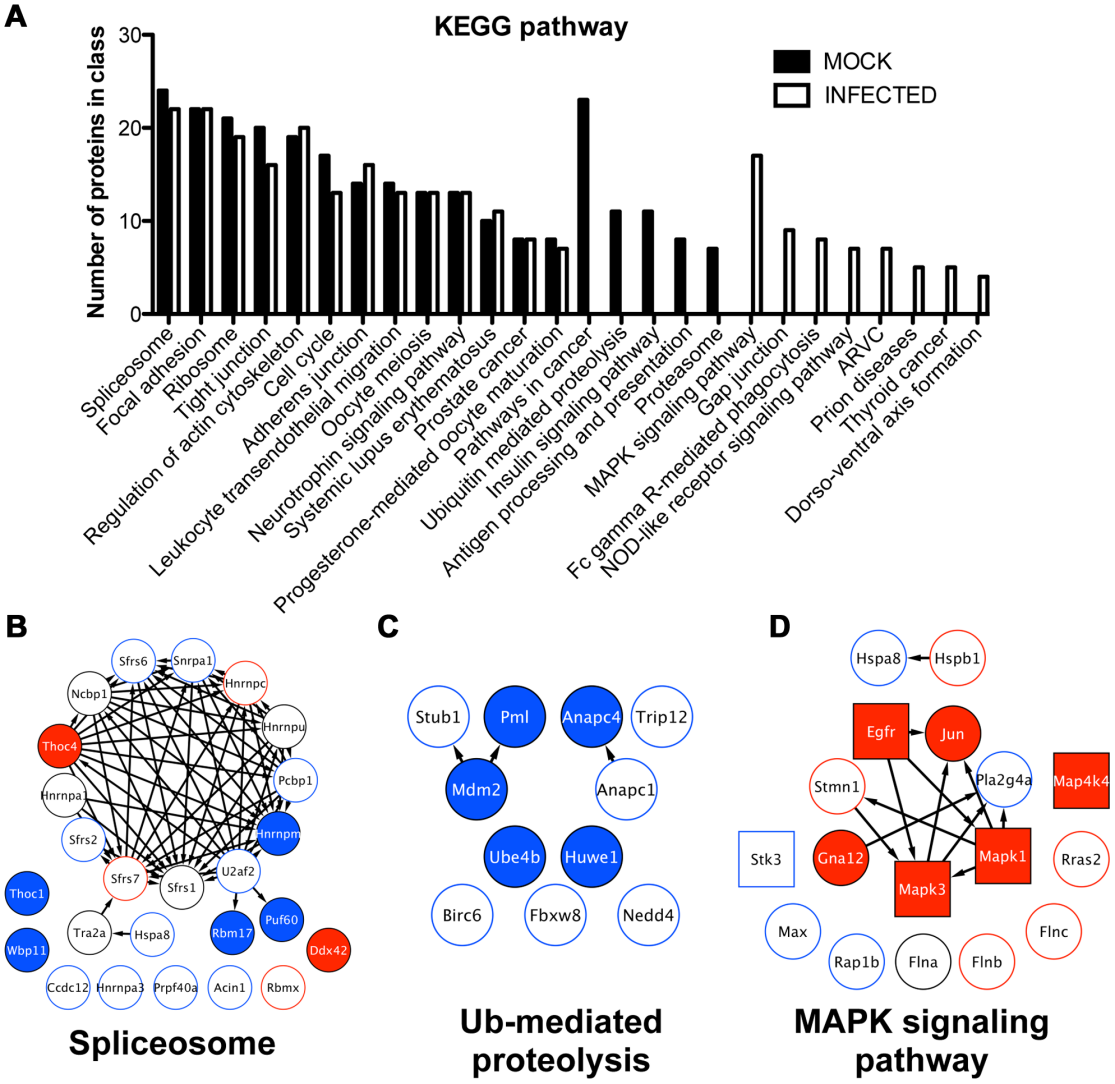
Identification of functional protein modules with altered phosphorylation patterns during MHV68 infection. (A) Phosphoproteins in control and infected phosphoproteomes were analyzed using DAVID, and KEGG pathways were identified. (B, C, and D) Individual proteins annotated to the selected KEGG pathways were extracted from the global phosphoprotein networks shown in Figures 4 and 5. Examples of common KEGG pathways (B), pathways absent in infection (C), and pathways represented only in infected cells (D) are shown. Solid red denotes detection only in infected cells. Solid blue denotes detection only in control cells. Red borders denote increased abundance during infection. Blue borders denote reduced abundance during infection. Black borders indicate no infection-related change in protein intensity. Gene names for each protein are shown. Kinases are depicted as square nodes.

We next performed a comparative analysis of Kyoto encyclopedia of genes and genomes (KEGG) pathways [64] that were represented in control and infected phosphoprotein networks (Fig. 6A). This analysis revealed a high degree of overlap between mock and infected data sets for thirteen KEGG-defined pathways, although the majority of redundant pathways do contain several proteins whose presence or abundance was influenced by infection. This point is illustrated by extracting proteins represented in the “spliceosome” KEGG pathway from the global phosphoprotein network. Five spliceosome proteins were absent from infected cells (solid blue), two were only detected in infected cells (solid red), and thirteen exhibited differing abundance between control and infected cells (blue or red outlines, respectively) (Fig. 6B). The phosphorylation status of only five spliceosome proteins remained unchanged. KEGG analyses also predicted several functional protein modules exclusively lost or induced during infection. For instance, control cells contained a high number of phosphoproteins annotated to the ubiquitin-mediated proteolysis pathway. Indeed, 5 of the 11 proteins represented were absent from infected cells, while the remaining 6 were less abundant during infection (Fig. 6C). Conversely, the MAPK signaling pathway was exclusively represented by infected phosphoproteins, including MAPK3/ERK1, MAPK1/ERK2, MAP4K4, c-Jun, and epidermal growth factor receptor (Fig. 6D). These data indicate that MHV68 infection modulates the phosphorylation status of specific functional protein modules despite a seemingly global redistribution of ontologically grouped protein phosphorylation.

### Elucidation of infection-related kinase signatures

**Figure 7:**
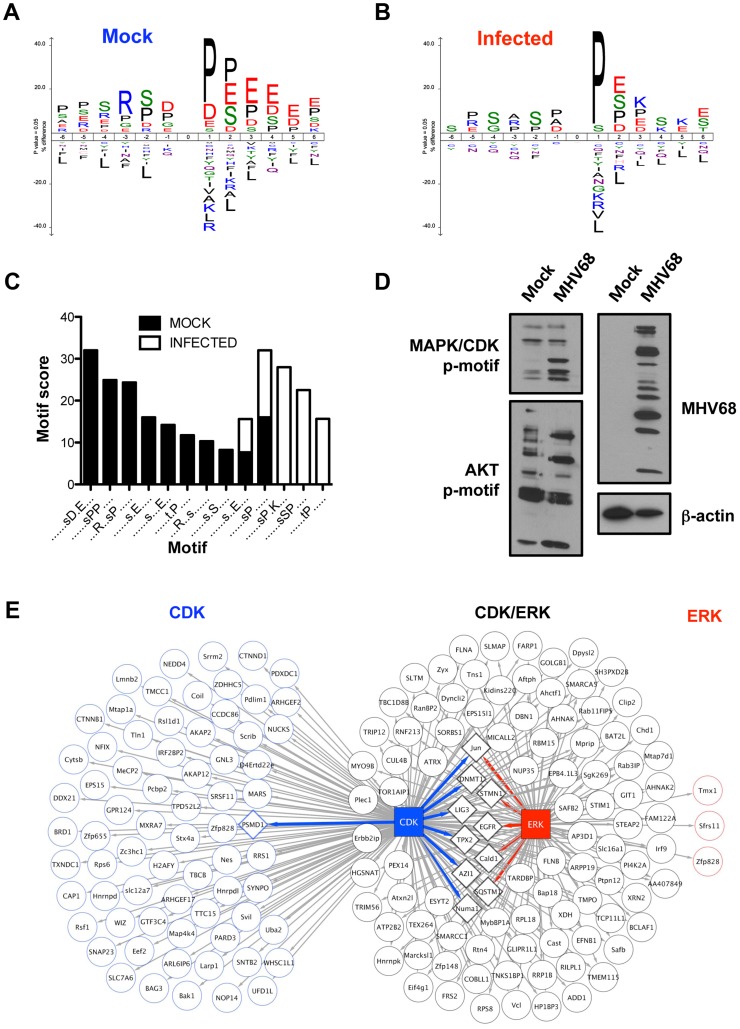
Comparative phosphorylation motif analyses reveal an infection-associated MAPK/CDK signature. Unique phosphopeptides present in either mock-infected (A) or infected cells (B) were analyzed using ICE-LOGO. Weighted sequence context for residues flanking either p-Ser or p-Thr at position 0 is provided where a larger size designation corresponds to increased relative abundance of a particular amino acid in the global data set compared to other residues at the same position. (C) Unique phosphopeptides were analyzed using Motif-X to identify motifs over-represented relative to the *Mus musculus* background proteome at a p-value of <0.000001. (D) 3T3 fibroblasts were mock infected or infected with MHV68 at MOI = 5 PFU/cell. Cells were harvested 18 h post-infection, and proteins were resolved by SDS-PAGE. Immunoblot analyses were performed using antibodies directed against the indicated phosphorylation motifs or proteins. (E) Putative CDK1/2 and ERK1/2 phosphorylated proteins present in infected cells were identified by GPS 2.1 analysis on high confidence settings. A separate STRING analysis was performed to identify proteins that functionally interact with either CDK1/2 or ERK1/2. Arrows connect the kinase to its predicted substrate. STRING-defined substrate interactions are depicted as diamond-shaped nodes, where kinase-substrate interactions are color-coded blue or red to denote CDK or ERK connectivity, respectively.

Distinct protein kinases phosphorylate specific amino-acid motifs present on target proteins. To determine if distinct phospho-motifs were overrepresented in the infection-associated data set relative to control identities, we segregated all individual phosphopeptides according to their presence or absence within control or infected cells. Sequence logos generated for each data set using the ICE-LOGO resource (http://iomics.ugent.be/icelogoserver/logo.html), a weighted representational analysis, demonstrate general differences in phosphorylated sequences between control and infected systems (Figs. 7A and 7B, respectively). Distinct phospho-motifs in each data set were identified using the Motif-X algorithm [65] (Fig. 7C). Interestingly, only two shared phospho-motifs were overrepresented in both control and infected phosphopeptides, S*XXE and XS*PX (Fig. 7C), where the asterisk designates the phosphorylated residue, relative to the background *Mus musculus* phosphoproteome. Control phosphopeptides exhibited more promiscuity in motif representation, with eight specific phospho-motifs identified as being enriched. A number of motifs present in control peptides were characterized by acidic residues downstream and an Arg upstream of the phospho-acceptor, including an AKT-related RXXS* motif (Fig. 7C). In contrast to control peptides, only three unique phospho-motifs were enriched during MHV68 infection (Fig. 7C). Each of these exhibited Pro-directed phospho-acceptors reminiscent of CDK and MAPK target sequences, including a classic CDK motif characterized by a basic residue at the -2 amino acid position relative to the phosphorylated residue [66]. Of note, detection of phosphorylated CDK1 and CDK2 was reduced during infection compared to control samples (Table S1). However, the CDK1/2 phosphorylations detected in our MS analyses correspond to *deactivating* the post-translational modification [67], [68]. Hence, a relative decrease in abundance within infected cells corresponds to CDK1/2 *activation* during infection, which was previously documented during productive MHV68 infection [69]. Additionally, the ERK1/2 phosphopeptides detected are indicative of activation (Table S1) [70]. Thus, the infection-associated phosphoproteome exhibits a strong CDK/MAPK phosphorylation signature, which differs from that of uninfected cells. This finding is in agreement with the identification of activated ERK1/2 and CDK1/2 by MS during infection and KEGG biological pathway predictions.

To directly test these predictions, we performed comparative immunoblot analyses using phospho-motif-specific antibodies directed against the AKT RXXS* target motif or MAPK/CDK XS*P/S*PXK motifs following mock-infection or infection with MHV68 (Fig. 7D). Although two unique RXXS*-containing proteins were prominent in infected cells, infection resulted in a general reduction in the number of detectable AKT-phosphorylated proteins. In contrast, infection resulted in enhanced detection of MAPK/CDK motif-containing proteins (Fig. 7D). As was the case with general phosphoprotein immunoblotting (Fig. S1), detection of MAPK/CDK phospho-motifs increased over time during infection (Fig. S6), which is consistent with the observation that ERK1/2 activation occurs during the early-to-late phase of the MHV68 replication cycle (See Fig. 3). These data independently verify the motif-based bioinformatics prediction that infection substantially alters the cellular signaling network, revealing that MAPK/CDK-related phosphorylation events are predominant in the infected system, while other signaling pathways apparently are repressed. These findings strongly suggest prominent roles for CDK and/or ERK signaling during MHV68 infection.

To gain insight into specific host proteins potentially regulated by ERK1/2 and/or CDK1/2 activity during infection, we performed high-confidence group-based phosphorylation scoring (GPS) analyses [71] to define infection-specific phosphopeptides that contain ERK1/2 and CDK1/2 motifs. The GPS analysis data table includes predictions for all available kinases for infection-specific phosphopeptides (Table S3), although other kinase-motif predictions are not discussed here. GPS analysis predicted 220 unique phosphopeptides derived from 171 host proteins that contain CDK1 and/or CDK2 motifs and 150 unique phosphopeptides derived from 103 host proteins for ERK1 and/or ERK2 (Fig. 7E and Table S3). 98 of the 253 predicted targets contained high-confidence motifs for both CDKs and ERKs, while 155 proteins were distinct targets – 150 CDK and 5 ERK (Fig. 7E). STRING analyses identified only 11 of these proteins as CDK or ERK interactors (Fig. 7E). Remarkably, of the 23 most abundant infection-specific host phosphoproteins illustrated in Figure 2D (not including ERK1 or ERK2), 18 contain GPS-predicted phosphorylation events on CDK and/or ERK target motifs. Of these, only c-Jun was a STRING-defined interaction partner for both CDKs and ERKs, which highlights it as a prioritized candidate for functional studies. These data strongly support the hypothesis that CDK and ERK-related signaling are predominant during productive MHV68 infection and further suggest that this serves to regulate a core set of proteins in the cellular phosphoprotein signaling network.

### Defining the function of CDKs, ERKs, and c-Jun in MHV68 lytic replication

**Figure 8:**
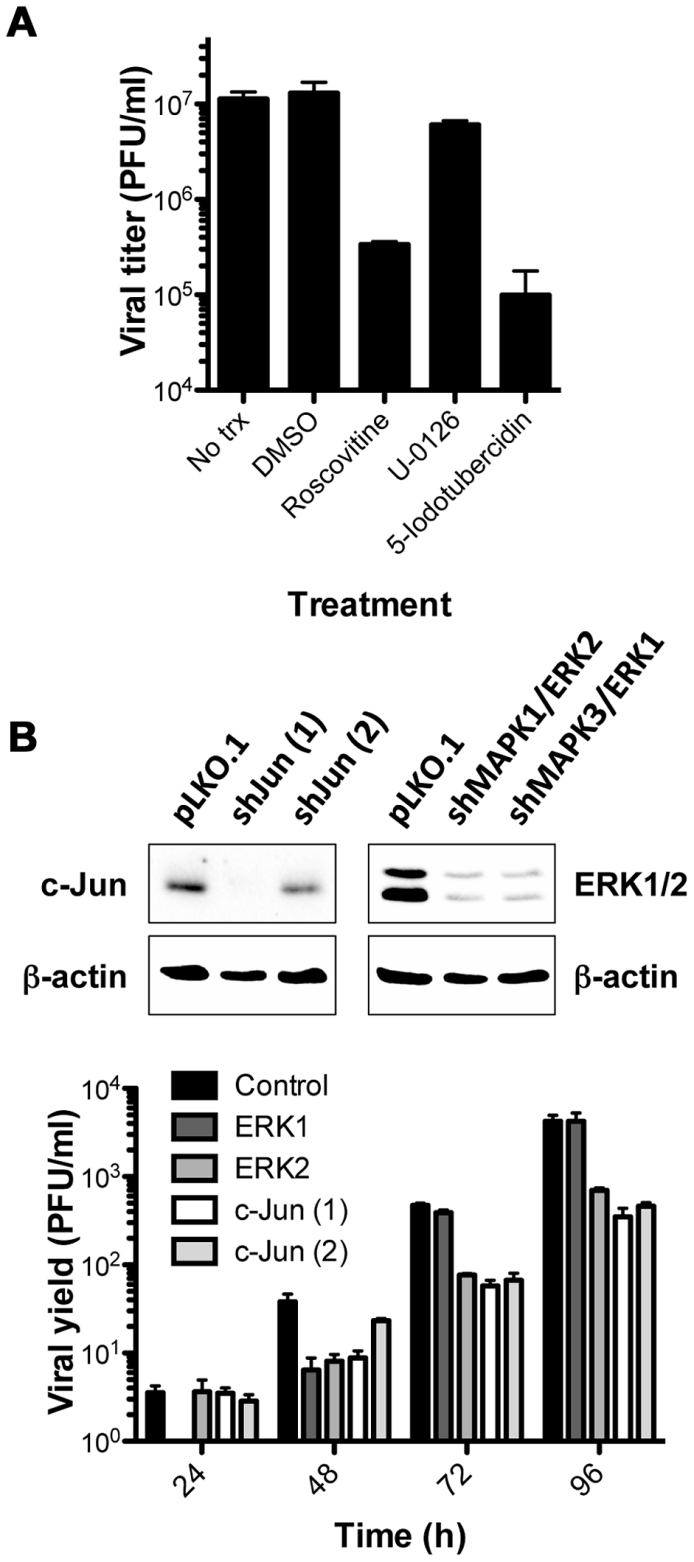
CDKs, ERK1/2, and c-Jun are required for efficient MHV68 replication. (A) 3T3 fibroblasts were treated with vehicle (DMSO), roscovitine, U0126, or 5-iodotubercidin at 10 µM concentration prior to infection with MHV68 at MOI = 10 PFU/cell. Cells were harvested 24 h post-infection, and viral titers were determined by plaque assay. Results are means of triplicate samples. Error bars represent standard deviations. (B) 3T3 fibroblasts were transduced with empty LKO-derived lentivirus or shRNA-encoding lentiviruses to knockdown each of the indicated host proteins. Immunoblot analyses to detect the indicated proteins were performed on cell lysates to evaluate the efficiency of shRNA knockdown. Depleted cells were infected with MHV68 at MOI = 0.05 PFU/cell, and cells were harvested at the indicated times post-infection. Viral titers were determined by plaque assay. Results are means of triplicate samples. Error bars represent standard deviations.

Three independent bioinformatics analyses strongly predict the importance of CDK and/or MAPK activity in productive MHV68 replication. First, GO and KEGG pathways analyses reveal an over-representation of proteins annotated to MAPK signaling present in the infected cell phosphoproteome. Second, infected cells exhibit a MAPK/CDK kinase motif signature. Finally, a very high percentage of infection-specific host phosphoproteins contain strong CDK and/or ERK phospho-acceptor motifs. To directly test whether CDK and ERK activity control MHV68 replication, we evaluated the capacity of pharmacologic inhibitors of CDK and ERK activity to block MHV68 replication in single-step growth curves. Target cells were pretreated with the CDK inhibitor roscovitine [72], [73], MEK inhibitor U-0126 [74], or ERK inhibitor 5-iodotubercidin [75], [76] prior to infection with MHV68, and viral titers were evaluated by plaque assay at 24 h post-infection. Compared to vehicle and untreated control infections, both roscovitine and 5-iodotubercidin treatments led to greater than 60-fold reduction in output titers. However, U-0126, which acts on MEK kinases 1 and 2 upstream of ERK activation [74], only minimally affected virus production (Fig. 8A). This suggests that MHV68-induced ERK activation occurs independent of MEK1/2. These data provide biological evidence that MHV68 usurps host CDK and ERK kinases for productive replication.

While roscovitine is a highly specific CDK inhibitor [72], [73], 5-iodotubercidin is a more promiscuous kinase inhibitor also capable of inhibiting adenosine monophosphate kinase and haspin [77], [78]. Highly specific chemical inhibitors of ERK activity are not currently available [79]. To more definitively evaluate roles in MHV68 replication, we utilized shRNAs targeting either ERK1 or ERK2 to knockdown ERK expression in 3T3 fibroblasts. At the same time, we also knocked down c-Jun expression in an effort to establish a possible downstream target of CDKs and/or ERKs required for viral replication. Compared to control shRNA knockdown cells, all of the shRNAs tested influenced the efficiency of MHV68 replication on some level (Fig. 8B). Interestingly, shRNAs targeting ERK1 slightly delayed the onset of viral replication (see 24 h and 48 h timepoints), but did not significantly reduce titers at later timepoints. In contrast, knockdown of c-Jun and ERK2 expression led to an overall reduction in output titers from 48–96 h post-infection, approximating 10-fold less efficient viral yield by 96 h post-infection (Fig. 8B). It is notable, however, that inhibition of viral replication was not absolute, but rather manifested as a relative deficit over time. This result may be a consequence of incomplete knockdown of the targeted proteins, or it also is possible that c-Jun, ERK1, and/or ERK2 function to enhance the efficiency of MHV68 replication, but are not absolutely required. It is worth noting that each of 4 unique shRNA constructs targeting either ERK1 or ERK2 reduced the expression of both ERK isoforms recognized by the antiserum used to evaluate knockdown efficiency (not shown). We reason this effect stems from the presence of a long stretch of highly homologous nucleotide sequence present in both isoforms. Thus, as immunoblot data suggest, it is likely that both ERK isoforms were depleted in these experiments, which complicates direct functional interpretations for particular ERK isoforms. Nonetheless, in conjunction with pharmacologic inhibition data, these findings provide strong evidence in support of the bioinformatically-predicted hypothesis that CDK and MAPK signaling promote MHV68 replication.

### Identification of v-cyclin as a viral factor that regulates phosphorylation during infection

**Figure 9:**
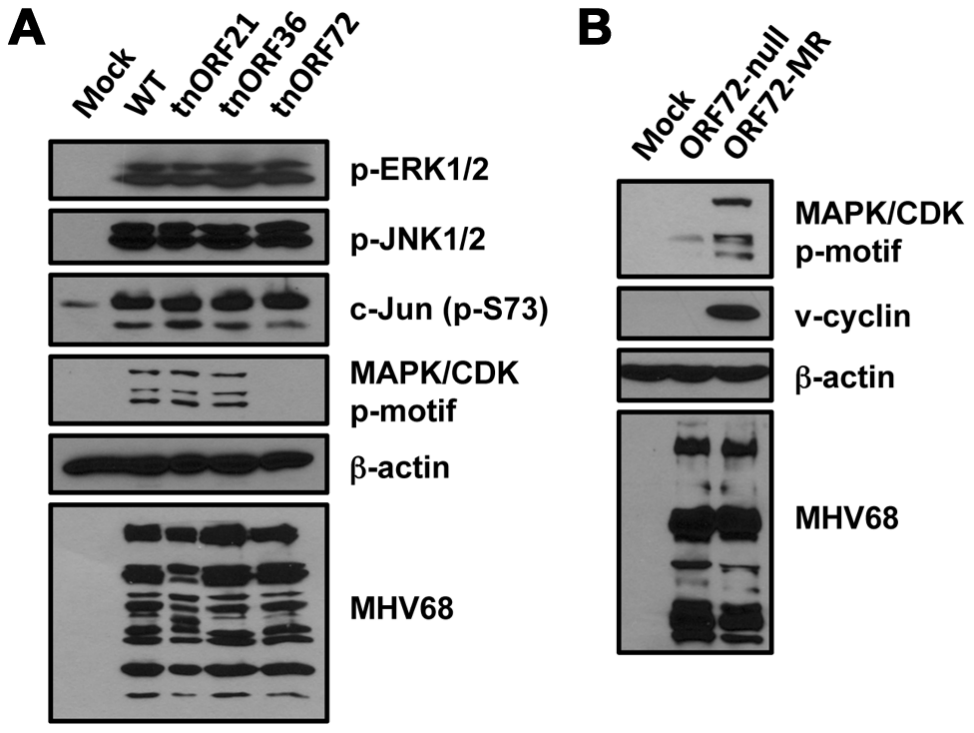
MHV68 D-type cyclin ortholog enhances CDK-related phosphorylation in infected cells. (A) 3T3 fibroblasts were mock infected or infected with WT or transposon mutant (tn) MHV68 viruses at MOI = 5 PFU/cell. (B) Cells were mock infected or infected with v-cyclin-null (ORF72-null) or genetically repaired WT control (ORF72-MR) MHV68 recombinant viruses. Cells were harvested 18 h post-infection, and proteins were resolved by SDS-PAGE. Immunoblot analyses were performed using antibodies directed against the indicated phosphorylated residues or proteins.

While the bioinformatics approaches employed above facilitated the identification of host molecules involved in GHV replication, it was not yet clear whether viral signaling proteins also contributed to infection-associated changes in the cellular phosphoprotein network. MHV68 encodes two kinases (ORF21 and ORF36) and an ortholog of cellular D-type cyclins (v-cyclin) capable of stimulating CDK activity (ORF72) [30], [69]. We therefore evaluated the capacities of WT MHV68, and transposon mutant viruses in which ORF21, ORF36, and ORF72 had been disrupted [80] to elicit phosphorylation of ERK1/2, c-Jun, and MAPK/CDK-motif containing proteins during infection. In comparison to mock-infected cells, all of the viruses tested potently induced ERK1/2 and c-Jun phosphorylation (Fig. 9A). However, the ORF72 transposon mutant did not elicit robust phosphorylation of MAPK/CDK-motif-containing proteins, while the other viruses did (Fig. 9A), thus provisionally identifying v-cyclin as a viral molecule that contributes to the infection-associated phosphorylation signature. As a more discrete test of this hypothesis, we infected cells with a recombinant virus containing a targeted disruption of ORF72 (ORF72-null) and its genetically repaired WT control, ORF72-MR [81]. While ORF72-MR infected cells exhibited enhanced MAPK/CDK-motif phosphorylation relative to mock infection, the ORF72-null virus did not elicit robust MAPK/CDK-motif phosphorylation (Fig. 9B). Thus, these data indicate that v-cyclin is a pathogen-encoded molecule that plays a prominent role in directing infection-related phosphorylation events.

## Discussion

Data presented in this manuscript describe a first-of-its-kind global phosphoproteomic analysis of gammaherpesvirus infection. The results offer new insight into infection-driven alterations in cellular phosphoprotein networks and requirements for productive viral replication. Quantitative analyses suggest that the vast majority of detectable phosphopeptides are regulated during GHV infection, and the identification of more than 400 induced or repressed host phosphoproteins and 18 viral phosphoproteins substantially increases the knowledge base of proteins phosphorylated during GHV infection. Complementary biochemical, bioinformatic, and pharmacologic inhibition studies depict a virus-induced redirecting of host-phosphoprotein interaction networks and functional pathways, likely dictated by ERK and CDK host proteins and a virus-encoded D-type cyclin ortholog. Together, these data demonstrate the potential power of systems-level analyses to define critical signal transduction pathways usurped during intracellular pathogen replication.

### General observations

Our study identified a total of 405 proteins that were only detected in either control (266) or infected cells (144 – 22 viral and 122 host). One of the proteins absent from infected cells, PML, is subject to ubiquitin-mediated degradation during MHV68 infection [36], [37]. This is also true for EBV, HSV and HCMV [82]. For HSV and HCMV, PML degradation limits an intrinsic host response to infection that represses viral gene expression [83], [84]. It is intriguing to speculate that other phosphoproteins not detected during infection also are degraded in order to limit inhibitory host-cell responses to MHV68 infection. Of course, the lack of detection in our phosphoproteomic analyses may also reflect a simple loss of phosphorylation, perhaps through phosphatase activity or viral inhibition of an upstream kinase. Indeed, phosphorylated FOXK1 was differentially detected between control and infected cells, yet expression of FOXK1 protein actually remains unchanged during MHV68 infection (J.A.S. and J.C.F., unpublished result). Likewise, infection-specific detection of ERK1/2 and c-Jun was a result of induced phosphorylation, rather than enhanced expression. Having established the framework here, in next generation experiments we envision pairing global differential proteomics techniques, such as stable-isotopic labeling of amino acids in cell culture (SILAC), with phosphoprotein enrichment to simultaneously determine changes in protein expression levels with induction or repression of phosphorylation. Kinetic analyses that combine these approaches would enable a dynamic assessment of how GHVs manipulate host protein expression levels and phosphorylation-dependent signaling events to gain control of the host cell. Moreover, such combined approaches would readily lend themselves to comparative studies aimed at determining specific contributions of viral signaling proteins, like v-cyclin, or host kinases, such as CDKs and ERKs.

The extent to which infection alters the phosphorylation status of specific proteins is remarkable and is dramatically illustrated in the GO protein class and global network analyses presented in Figures 2D and Figures 4 and 5 respectively. Indeed, 86% of proteins we identified exhibited intensity changes of greater than two-fold. By comparison, analogous phosphoproteomic analyses suggest that fewer than 15% of phosphoproteins are differentially regulated during cellular responses to DNA damage or growth factor receptor signaling [12], [13], 24% in response to *Salmonella* infection of cultured cells [35], and 14% induced by HIV binding to cells [85]. We hypothesize that these comparative differences in phosphoprotein regulation reflect the extent to which an intracellular pathogen must usurp multiple host cell biosynthetic systems and evade innate immune detection during viral replication. As an obligate intracellular parasite, a herpesvirus must commandeer host cell machinery involved in transcription, DNA replication, nuclear import and export, translation, and vesicle transport, while limiting or redirecting cell death, antiviral, and immunomodulatory host-cell responses to infection. The finding that infection alters the composition of protein clusters within the host phosphoprotein network may provide insight into mechanisms by which GHVs, and perhaps intracellular pathogens in general, gain control of functional modules within the host cell to facilitate viral replication. We hypothesize that GO analyses further illustrate this point, revealing how infection-related phosphorylation appears directed toward proteins involved in distinct biological processes, such as nucleosome organization and ribosome or rRNA regulatory processes (Table S2). One might also hypothesize that the absence of phosphoproteins in specific GO classes or KEGG pathways during MHV68 infection, such as those involved in ubiquitination, signal transduction, and cell death (Table S2), illuminate host processes shut down by virus-directed events.

Along these same lines it is important to consider the possibility that some of the signaling events we observed reflect the host cell response to infection. Roles for kinases in propagating and enforcing antiviral responses have been extensively studied [55], [86], [87], [88], and recent systems-level analyses demonstrate broad reorganization of host cell transcription and signaling networks following exposure to immuno-stimulatory microbial products [89]. From this study and numerous others, functions of MAPKs clearly influence the host cell response to infection. Given the strong MAPK signature present during MHV68 infection, it is possible that, beyond ERK, JNK [24], or Tpl2/Cot1 [26], other MAPKs that do not overtly facilitate viral replication influence the phosphoprotein network as part of the innate host-cell response to infection. Thus, it will be of interest to elucidate if and how other MAPKs or unrelated innate-immune kinases influence the infection-associated phosphoproteome.

### Usurping ERK and CDK signaling pathways to facilitate viral replication

A key feature of the MHV68 phosphoproteome is that it offers direct insight into specific host signaling pathways usurped by MHV68 to facilitate infection. The data also highlight several interesting parallels with other herpesviruses. The concurrence of several independent bioinformatics analyses in highlighting the prominence of ERK/MAPK and CDK-related phosphorylation during MHV68 infection was impressively emphasized by the detection of ERK/CDK motif phosphorylation on 55% of infection-specific host phosphoproteins (Table S3, compare to Table S1). Functional tests using pharmacologic inhibitors and shRNA knockdown confirmed the importance of CDK and ERK signaling in MHV68 replication (Fig. 8). With regard to ERK, a number of previous studies have demonstrated presumably biphasic roles for ERK in the KSHV lytic replication cycle. In the first phase, ras/raf-MEK-ERK signaling pathways are capable of promoting reactivation from latent infection by promoting immediate-early viral gene expression [38], [39], [40]. As one would expect given the involvement of MEK in this canonical pathway, this phase of ERK activation – and consequently KSHV reactivation downstream of ras/raf, MEK, or chemical induction with TPA – is inhibited by treatment with the pharmacologic MEK inhibitor U-0126 [38], [39], [40]. In contrast, a second phase of ERK activation during the KSHV lytic cycle is mediated by ORF45, a multifunctional tegument protein that stabilizes a ternary complex composed of ORF45, ERK, and p90 ribosomal S6 kinase [43], [90]. In agreement with the finding that MHV68 replication (Fig. 8) and ERK activation (not shown) were not inhibited by U-0126 treatment, ORF45-directed ERK activation also is insensitive to MEK inhibition [43], leading us to hypothesize that MHV68 ORF45 may similarly promote ERK activation. Indeed, MHV68 and KSHV ORF45 proteins are functionally interchangeable in facilitating viral replication [91]. Hence, in addition to enhancing a general understanding of ERK functions in GHV infection, the identification of previously unknown putative ERK-phosphorylated proteins within infected cells may provide new insight pertaining to ORF45-directed enhancement of viral gene expression, translation, and viral egress [92], [93], [94]. It will also be of interest to determine if predicted ERK phosphorylation sites we identified on ORF45 (Table 1 and Table S3) are bona fide ERK targets, and whether they exert functional control over ORF45 complex formation during viral replication.

As a group, herpesviruses are thought to usurp CDK signaling in order to provide an S-phase-like environment amenable to replicating the viral DNA genome. For instance, reactivating EBV drives high S-phase cyclin expression and CDK activity, while at the same time inhibiting host DNA replication [95], possibly through induction of a DNA damage response (DDR) [96] and/or inactivation of the MCM4-6-7 helicase complex [97]. Further and in agreement with our data, pharmacologic inhibition of CDK activity with roscovitine also inhibits lytic replication of EBV [98], HCMV [99], [100], and HSV1 [101], [102], which strongly suggests that usurping cyclin/CDK activity is a universal requirement of herpesviruses. However, roles for CDK activity in promoting GHV replication have been minimally explored. And, although a few EBV targets of cyclin B/CDK1 are known [103], information as to host substrates of cyclin/CDK activity during lytic GHV replication are lacking. In this regard, identification of potential CDK-phosphorylated proteins during MHV68 infection may reveal how GHVs direct CDK activity to foster efficient viral replication.

A related question asks which viral factors contribute to the CDK phosphorylation signature during lytic GHV replication. As a homeostatic cellular process, cyclin/CDK activity is tightly controlled on several levels. This includes transcriptional regulation of cyclin genes, phosphorylation-dependent activation and inactivation of CDKs, and direct inhibitory interactions of cyclin/CDK holoenzyme complexes with CDK inhibitors (CKIs), such as p21 and p27 [67]. At the host transcriptional level, both MHV68 and KSHV LANA proteins induce transcription of cellular cyclin genes [104], [105]. Although the functional significance of LANA-mediated induction of host cyclins during productive gamma-2-herpesvirus replication has not been specifically tested, it is interesting to note that LANA-null MHV68 exhibits attenuated replication both in culture and in vivo [106], [107] that is dependent on its transcriptional regulatory capacity [108]. Additionally, conserved herpesvirus protein kinases (CHPKs) in GHVs and beta-herpesviruses, which are required for efficient viral replication [11], [27], [109], [110], exhibit CDK-like functions, most notably pRb phosphorylation and the capacity to complement temperature sensitive yeast *CDC28* (*S. cerevisiae* CDK ortholog) mutants for growth [111], [112]. Further, BGLF4, the EBV CHPK, exhibits partially overlapping substrate specificity with cyclin B/CDK1 in vitro [103].

Finally, gamma-2-herpesviruses, including MHV68 and KSHV, encode an ortholog of cellular D-type cyclins [29], [30]. Viral cyclins exhibit an expanded capacity to interact with host CDKs [69], [113], [114] and are resistant to inhibition by CKIs [115]. Indicative of their capacity to stimulate cell-cycle progression [116], [117], v-cyclins are oncogenic when expressed in mice as a transgene [116], [118], [119], and MHV68 v-cyclin is singularly required for pRb phosphorylation during lytic MHV68 infection [69]. Further, the KSHV cyclin ortholog is thought to play initiating and sustaining roles in KSHV-related cellular transformation [33], [120], [121]. The finding that cells infected with v-cyclin-null MHV68 exhibit reduced MAPK/CDK phosphorylation (Fig. 9) strongly suggests that v-cyclin is a major contributor to the MAPK/CDK signature of lytic MHV68 infection. Although v-cyclin is not absolutely required for viral replication in cell culture, v-cyclin-null MHV68 exhibits attenuated acute replication, delayed latency establishment, and a severe reactivation defect *in vivo* [81], [122]. An elegant study using recombinant MHV68 viruses in which v-cyclin was exchanged with cellular cyclins A, D, or E demonstrates overlapping or redundant functions for host and v-cyclins in some, but not all, aspects of MHV68 pathogenesis [123], which may explain why v-cyclin is not necessary for MHV68 replication in culture [81], but roscovitine potently blocks viral replication (Figs. 8 and 9). Moreover, v-cyclin expression is necessary for MHV68 transformation of primary B cells in culture [124] and lymphoproliferative disease and lethal pneumonia in vivo [125], [126]. While further validation clearly is necessary, it is tempting to speculate that the CDK-motif containing proteins identified in this report are critical host-cell targets of v-cyclin that influence GHV pathogenesis.

### Concluding remarks

Together, the data presented herein enhance our understanding of the GHV-host interaction. In addition to defining new proteins and hypotheses for experiments to foster a more complete understanding of basic mechanisms of GHV replication and pathogenesis, the identified ERK and CDK-predicted phosphoproteins may encompass new host targets for therapeutic interventions. Our data strongly support the further evaluation of ERK and CDK inhibitors as treatments for lytic cycle-associated GHV diseases, such as IM or KS. Toward defining the pathogen-host interaction in general, it will also be of interest to determine whether global reorganization of the host phosphoprotein network is a phenotype shared with unrelated intracellular pathogens, such as RNA viruses or bacteria. If so, are common signaling pathways or macromolecular complexes targeted? And, could these common pathogen-exploited host proteins serve as novel candidates for new generalized treatments? The approaches we describe should be readily adaptable to other systems. Thus, our studies lay the foundation for future comparative analyses of this sort, as well as for defining differences and commonalities between *de novo* lytic GHV replication and reactivation, or comparative studies with alpha and beta-herpesviruses.

## Materials and Methods

### Ethics statement

Mouse experiments performed for this study were carried out in accordance with NIH, USDA, and UAMS Division of Laboratory Animal Medicine and IACUC guidelines. The protocol supporting this study was approved by the UAMS Institutional Animal Care and Use Committee (Animal Use Protocol 3270). Mice were anesthetized prior to inoculations and sacrifice to minimize pain and distress.

### Cell culture and viruses

Swiss-albino 3T3 fibroblasts (referred to as 3T3 fibroblasts throughout) were purchased from ATCC. All cells were cultured in Dulbecco's modified eagle medium supplemented with 10% fetal calf serum (FCS), 100 units/ml penicillin, 100 µg/ml streptomycin, and 2 mM L-glutamine (cMEM). Serum starvation involved culturing of cells in media containing 0.5–1% FCS for 18–24 hours prior to infection or treatment. Cells were cultured at 37°C with 5% CO_2_ and ∼99% humidity. Wild-type MHV68 was strain WUMS (ATCC VR1465), WT BAC-derived MHV68 [127], or BAC-derived MHV68-YFP [128]. *ORF50*-null MHV68 (50.STOP) was previously described [52]. UV-inactivation of WT MHV68 was accomplished by diluting virus stock to 1×10^7^ PFU/ml and autocrosslinking in 60 mm plates using a Stratalinker prior to infection. Disruption of viral gene expression was confirmed by immunoblot analyses to detect viral proteins. Cells were infected by low-volume adsorption of viruses to the monolayer. The time of adsorption for all experiments was considered t = 0. Inocula were removed after 1 h, and cells were cultured in a normal volume of serum starvation medium.

### Peptide preparation

1×10^7^ mock-infected or infected cells were harvested 18 h post-infection by scraping in cold phosphate-buffered saline (PBS). Cells were pelleted at 700 g for 5 min, and snap frozen in liquid N_2_. Cell pellets were lysed on ice in 500 µL buffer containing 50 mM Tris (pH 7.5), 50 mM NaCl, 0.05% surfactant (Promega) supplemented with protease and phosphatase inhibitor cocktails (Thermo Scientific) with vortexing every 5 to 10 min for 30 min. Insoluble debris was removed by centrifugation at 11000 g for 11 min. Protein concentration in the resulting solution was determined by BCA assay. 1 mg of protein in solution was concentrated by centrifugation through a 3 kDa filter (Amicon) and rinsed with 500 µL buffer containing 0.025% surfactant and 25 mM ammonium bicarbonate (ABC). The concentrated protein mixture was then diluted to 900 µL total volume in 25 mM ABC. Proteins were reduced in 5 mM DTT for 20 min at 60°C, followed by alkylating in the dark with 25 mM iodoacetamide for 30 min at 25°C. Buffer exchange to 25 mM ABC was performed by centrifugation through 3 kDa filters and the resulting concentrate was diluted to 900 µL total volume in 25 mM ABC. Trypsin diluted in 0.01% trifluoroacetic acid (TFA) was added to the protein mixture (1∶50 w/w) and incubated overnight at 37°C. Digests were quenched with 0.1% TFA. A 20 µL aliquot of the quenched trypsin digest was set aside for analysis.

### Phosphopeptide enrichment

Phosphopeptide enrichment was based on a previously described method [129]. Digested peptide samples (from 1 mg total protein) were desalted using Sep-Pak columns. Sep-Pak columns were primed with a 75/25 mixture of buffers B/A (Buffer A - 2% acetonitrile (ACN), 0.1% formic acid; Buffer B - 75% ACN, 0.1% formic acid) and rinsed with 2 mL Buffer A. Peptide samples were passed through Sep-Pak columns, followed by rinsing with 2 ml Buffer A. Peptide were eluted with 75/25 Buffer B/A mixture and desiccated in a speed vac. TiO_2_ beads were pre-incubated in Loading Buffer 1 (LB1 – 65% ACN, 2% TFA, saturated glutamic acid) at a ratio of 1 mg beads to 20 µl LB1. 10 µl of TiO_2_ bead slurry was added to each desalted peptide sample and agitated for 10 min. Beads were collected by centrifugation at 3000 rpm for 30 sec, and the enrichment was repeated twice more with a fresh aliquot of TiO_2_ beads for each peptide solution. Thus, three successive enrichments were performed for each sample. Beads and phosphopeptides were washed three times for 10 min with agitation using 800 µl Wash Buffer 1 (65% ACN, 0.1% TFA), followed by 3 identical washes with 800 µl Wash Buffer 2 (65% ACN, 0.5% TFA). Phosphopeptides were eluted by incubation in Elution Buffer 1 (300 mM NH_4_OH, 50% ACN) for 10 min with agitation, followed by identical treatment with Elution Buffer 2 (500 mM NH_4_OH, 60% ACN). Eluted phosphopeptides were desiccated in a speed vac.

### Mass spectrometry

Peptide samples acidified to 0.1% formic acid final concentration were analyzed by nano-LC/MS/MS technique on an ion trap tandem mass spectrometer (MS). An auto-sampler was used for automatic injection of tryptic peptides from a 96 well plate to the NanoLC 2D system (Eksigent). Peptides were separated by reverse phase HPLC using a 10 cm long analytical column (C12 resin, Phenomenex). HPLC eluate was ionized by ESI (Electrospray ionization), followed by MS/MS analysis using collision induced dissociation on an LTQ Orbitrap hybrid MS (Thermo Finnigan, San Jose, CA) with two mass analyzers - Linear ion trap (LTQ), and Orbitrap. One MS scan by Orbitrap was followed by 7 MS/MS scans by LTQ. Other relevant parameters include - spray voltage 2.0 kV; m/z range of 350–1500; isolation width (m/z) of 2.5; and normalized collision energy 35%. MS spectrum data were acquired using XCalibur 2.0 software. MS technical information provided in Table S4. Raw data files are available at https://chorusproject.org/anonymous/download/experiment/-7729244682105805562.

### Data analysis

Data analysis was performed using MaxQuant 1.0.12.31 [130]. Experiment design consisted of two sample types, ‘mock’ (Expt1) and ‘infected’ (Expt2). Each sample type had two biological duplicates (A and B) and three technical replicates (1, 2 and 3). Therefore we had 12 MS data files, one for each MS run, namely: M1A, M1B, M2A, M2B, M3A, and M3B for mock samples and corresponding Mr1A, Mr1B, Mr2A, Mr2B, Mr3A, and Mr3B for infected samples. MS/MS peaks were searched against a concatenated forward and reversed version of IPI_mouse_v3.82 [131] database using the Mascot 2.2 search engine [132] via MaxQuant. False discovery rate for identification was less than 1% as estimated by the number of hits to the reversed sequences in the decoy database. Additional technical parameters are provided in Table S4. Thus, we identified a total of 986 proteins at 1% FDR. These included 791 phosphoproteins with 1101/2271 unique phospho (ST) and 38/39 unique phospho (Y) site positions.

Proteins differentially enriched between mock and infected samples were identified by (1) present-absent call based on peptide intensity (zero intensity was considered as absent call) and (2) 1.5-fold increase or decrease in intensity ratio of infected/mock. This analysis was performed using the protein intensities from the MaxQuant output file proteinGroups.txt. The complete quantitated data set is provided in Table S1.

### Bioinformatics analyses

Version 9.05 of the STRING resource [59] was used to generate protein interaction networks for MS-identified proteins. STRING networks are provided in Figures S4 and S5. All interactions are predicted with medium confidence threshold of 0.400, and all active predictive methods were allowed. Interaction networks were processed in Cytoscape 2.8 [60] to assign integer values and color coding to visually depict presence, absence, increase or decrease in protein intensity during infection. Disconnected nodes are not included in the Cytoscape output. Biological Process gene ontology analyses were performed using the BiNGO cytoscape plugin [63]. Overrepresented categories were identified relative to the *Mus musculus* background gene set using a hypergeometric test with Benjamini and Hochberg false discovery rate correction to define significance. Sorted data provided in Table S2. Clustered proteins in phosphoprotein networks were identified using the MCODE cytoscape plugin [62]. Protein Class gene ontology analyses were performed using PANTHER 7.2 [133] against the *Mus musculus* background gene set. Kyoto encyclopedia of genes and genomes (KEGG) pathways enrichment was defined through DAVID [134], [135]. For Motif-X [65] analyses, unique phosphopeptides for either mock or infected samples were identified from the global phosphopeptide sequence list. Unique phosphopeptides for either data set were identified using the IPI mouse proteome as background with a minimum of 20 occurrences per motif and a significance threshold of 0.000001. 13 amino acid long motifs were defined where the phosphorylated residue is at position 7. Shorter peptides were extended from mouse IPI database. Prealigned Motif-X output text files were used to generate global unique sequence logos in ICE-LOGO (http://iomics.ugent.be/icelogoserver/logo.html). Group-based Prediction Systems (GPS) 2.1 software [71] was utilized at highest-threshold setting to perform batch identifications of phosphopeptides containing specific kinase target motifs. Sorted data provided in Table S3.

### Pharmacologic inhibition and shRNA knockdown

Serum-starved 3T3 fibroblasts were untreated or pretreated with either DMSO (vehicle), U0126 (LC Laboratories), roscovitine (Cayman Chemical), or 5-iodotubercidin (Cayman Chemical) at 10 µM final concentration for 1 h prior to low-volume adsorption with MHV68 at MOI = 5 PFU/cell. Inocula were removed, and cells were cultured in a normal volume of medium. Cells were harvested 24 h post-infection, and progeny virions were liberated by freeze-thaw lysis. Viral titers were determined by MHV68 plaque assay as described [136]. A separate plate was harvested immediately after adsorption and subsequently titered to ensure that drug treatment did not inhibit viral attachment and to determine the 0 h titers for viral yield calculations.

Lentiviral pLKO.1-based shRNA vectors were purchased from Sigma. The following shRNA constructs were used in this study: TRCN0000229528 (c-Jun-1, NM_010591.2-2974s21c1), TRCN0000042693 (c-Jun-2, NM_010591.1-994s1c1), TRCN0000360511 (c-Jun-3, NM_010591.2-2270s21c1), TRCN0000023160 (MAPK1-1, NM_011949.2-490s1c1), TRCN0000054730 (MAPK1-2, NM_011949.2-921s1c1), TRCN0000023186 (MAPK3-1, NM_011952.1-305s1c1), TRCN0000023187 (MAPK3-2, NM_011952.1-662s1c1). Lentiviruses were produced by transfecting 293T cells with shRNA vector plasmid and packaging vectors pSPAX2 and pHCMV-G. Lentiviral supernatants were harvested at 48 and 72 hours post-transfection. Due to inefficient knockdown using single vectors, 3T3 fibroblasts were transduced in 24 hour succession with two distinct lentiviruses each targeting the specified protein. c-Jun (1) stable knockdown cells were transduced with shRNA vectors 1 and 3. c-Jun (2) stable knockdown cells were transduced with shRNA vectors 2 and 3. Transduced cells were selected with puromycin (4 µg/ml) and expanded. Stable knockdown cells were plated and infected with MHV68 at MOI = 0.05 PFU/cell. Cells were harvested at the indicated times and viral titers were determined by plaque assay [136]. Viral yields were determined by dividing output titers at the indicated timepoint by 0 h titers which represent input virus inoculum.

### Immunoblot analyses

Cells were lysed with alternative RIPA buffer (150 mM NaCl, 20 mM Tris, 2 mM EDTA, 1% NP-40, 0.25% DOC, supplemented with complete mini-EDTA free protease inhibitors (Thermo) and phosphatase inhibitor cocktail 2 (Thermo) and quantified using the Bio-Rad DC or Thermo BCA protein assay prior to resuspending in Laemmli sample buffer, or equivalent numbers of cells (1–2×10^5^) were directly lysed with 100 µl Laemmli sample buffer. Samples were heated to 100°C for 10 min and resolved by SDS-PAGE. Resolved proteins were transferred to nitrocellulose and identified with the indicated antibodies. ORF59, v-cyclin, and MHV68 antisera were previously described [69], [116]. ORF21 mAb was a gift from P.G. Stevenson. MHV68 antiserum was generated as previously describe [137]. P-Ser (AB1603), p-Thr (05-1923), and p-Tyr (05-321) antibodies were purchased from Millipore. P-c-Jun (S73-#9164), c-Jun (#9615), p-ERK1/2 (#4370), ERK1/2 (#4695), p-MARCKS (#2741), and S*PXK/PXS*P motif (#2325) RXXS/T* motif (#9614) antibodies were purchased from Cell Signaling Technology. β-actin mouse monoclonal antibody was purchased from Sigma (A2228). Immobilized antigen and antibody were detected with HRP-conjugated secondary antibodies and SuperSignal Pico West ECL reagent (Thermo Scientific) or Clarity ECL reagent (BioRad) and exposed to film or imaged on a BioRad ChemiDoc MP digital imaging system.

### Mouse infections and flow cytometry

Female C57BL/6 mice 6 to 8 weeks of age were purchased from the Jackson Laboratory. Mice were sterile housed in the animal facility at the University of Arkansas for Medical Sciences in accordance with all federal and university DLAM guidelines. Mice were mock-infected with intraperitoneal injection of 0.2 ml of DMEM or infected intraperitoneally with 10^6^ PFU of H2B-YFP virus diluted into 0.2 ml of DMEM. Four days post-infection mice were sacrificed by isoflurane overexposure and cervical dislocation. Spleens were harvested, homogenized into single-cell suspensions, and erythrocytes were lysed with red blood cell lysis buffer (Sigma) according to manufacturers instructions.

For flow cytometry, splenocytes were fixed and permeabilized with Foxp3/Transcription Factor Staining Buffer Set according to the manufacturers instructions (eBioscience, #00-5523-00). Fixed and permeabilized cells were washed twice with FACS buffer (3 µM BSA, 1 mM EDTA in PBS) and stained with PE-conjugated rabbit anti-c-Jun pS73 (Cell Signaling, #8752) and goat anti-GFP (Rockland, #600-101-215) antibodies diluted in FoxP3 wash buffer (eBioscience) for 30 minutes at room temperature. Stained cells were washed twice with FoxP3 wash buffer and incubated with donkey anti-goat secondary antibody conjugated to Alexa fluor 488 nm (Invitrogen, #A-11055) diluted in FoxP3 wash buffer for 30 minutes at room temperature. Stained splenocytes were washed twice with FoxP3 wash buffer and resuspended in FACS buffer. Antibody-stained cells were analyzed by flow cytometry using a Fortessa (Becton Dickinson) to quantify cellular YFP and c-Jun (p-S73) levels. Splenocytes from mock-infected animals were used to gate for YFP− and YFP+ cell populations, as infected (YFP+) splenocytes are absent from these animals. This gating strategy allowed for detection of YFP+ splenocytes, which expressed YFP at levels exceeding the prior gate.

## Supporting Information

Figure S1 **Identification of timepoints at which MHV68 elicits changes in phosphorylation patterns during productive infection.** 3T3 fibroblasts were mock-infected or infected with MHV68 at MOI = 5 PFU/cell. Cells were harvested at the indicated times post-infection, and proteins were resolved by SDS-PAGE. Immunoblot analyses were performed to detect infection-related differences in proteins phosphorylated on Tyr or Ser. 18 h timepoints (red box) were selected for label-free quantitative phosphoproteomic analyses.
(TIF)

Figure S2 **ORF59 is a viral phosphoprotein.** (A) Schematic representation of MHV68 ORF59 highlighting sequenced amino acids and phosphorylated residues in red. (B) 3T3 fibroblasts were mock-infected or infected with MHV68 at MOI = 5 PFU/cell. Cells were harvested 18 h post-infection, and ORF59 was captured by immunoprecipitation. Immunoprecipitates and total cell lysates were resolved by SDS-PAGE and transferred to nitrocellulose. Immunoblot analyses were performed to detect ORF59, phospho-Ser, and phospho-Thr. The asterisk corresponds to IgY heavy chain. The arrowhead corresponds to precipitated ORF59.
(TIF)

Figure S3 **Enhanced c-Jun S73 phosphorylation occurs specifically in infected cells.** (A and B) 3T3 fibroblasts were mock-infected or infected with H2B-YFP-expressing MHV68 (Collins and Speck, 2012) at MOI = 5 PFU/cell. 18 h post-infection cells were harvested and processed for intracellular antigen staining to detect H2B-YFP (A) or c-Jun phosophorylated on S73 (B) by flow cytometry. H2B-YFP expressing cells were identified by flow cytometry following staining with goat anti-GFP (Rockland Immunochemicals) and donkey anti-goat Alexa 488 (Invitrogen). H2B-YFP+ cells were identified relative to mock-infected cells. c-Jun (S73) intensities were determined in mock-infected and H2B-YFP+ cells by flow cytometry following staining with PE-conjugated rabbit anti-phospho-S73 c-Jun (Cell Signaling Technology). Isotype controls were used to determine background staining intensity (not shown). (C) 6–8 week-old C57BL/6 mice were intraperitoneally mock-inoculated or inoculated with 10^6^ PFU of H2B-YFP-expressing MHV68. 4 days post-infection, mice were sacrificed, and splenocytes were isolated and processed as in A and B to detect H2B-YFP and S73-phosphorylated c-Jun (See Figure 3). Shown are representative dot plots to demonstrate the relative percentage of H2B-YFP+ cells detected in infected animals and the gating strategy used to segregate YFP− and YFP+ cells for comparative analyses of c-Jun phosphorylation within the same animal. A total of 3 mock-inoculated and 4 H2B-YFP MHV68 inoculated animals were used for these experiments.
(TIF)

Figure S4 **STRING analyses predict phosphoprotein interaction networks for uninfected control cells.** Proteins identified in phosphoproteomic analyses were submitted to STRING (Szklarczyk et al., 2011) for bioinformatic predictions of phosphoprotein interaction networks. STRING-predicted total protein networks for mock-infected cells are shown. Networks are provided as confidence views where the edge weight (lines connecting nodes) correlates to confidence of the interactions. Interactions were predicted with standard STRING-defined confidence values of 0.400. STRING data where used to generate phosphoprotein networks presented in Figure 4.
(TIF)

Figure S5 **STRING analyses predict phosphoprotein interaction networks for MHV68-infected cells.** Proteins identified in phosphoproteomic analyses were submitted to STRING (Szklarczyk et al., 2011) for bioinformatic predictions of phosphoprotein interaction networks. STRING-predicted total protein networks for infected cells are shown. Networks are provided as confidence views where the edge weight (lines connecting nodes) correlates to confidence of the interactions. Interactions were predicted with standard STRING-defined confidence values of 0.400. STRING data where used to generate phosphoprotein networks presented in Figure 5.
(TIF)

Figure S6 **Identification of timepoints at which MHV68 elicits MAPK/CDK phosphorylation during productive infection.** 3T3 fibroblasts were mock-infected or infected with MHV68 at MOI = 5 PFU/cell. Cells were harvested at the indicated times post-infection, and proteins were resolved by SDS-PAGE. Immunoblot analyses were performed to detect infection-related differences in proteins phosphorylated on CDK/MAPK motifs (antibody from Cell Signaling Technology). Blots were also probed with MHV68 antiserum to demonstrate progression through the lytic replication cycle.
(TIF)

Table S1 **MaxQuant analyzed phosphoproteomic data.**
(XLS)

Table S2 **Gene ontology biological processes analysis of phosphoprotein networks.**
(XLSX)

Table S3 **GPS 2.1 predictions for infection-specific phosphopeptides.**
(XLSX)

Table S4 **Mass spectrometry technical information.**
(DOC)
