# Supplementary figures and images for: Phosphoproteomic Analyses Reveal Signaling Pathways That Facilitate Lytic Gammaherpesvirus Replication
Source: PLoS Pathog. 2013 Sep 19;9(9):e1003583. doi: 10.1371/journal.ppat.1003583 (PMC3777873; doi:10.1371/journal.ppat.1003583)

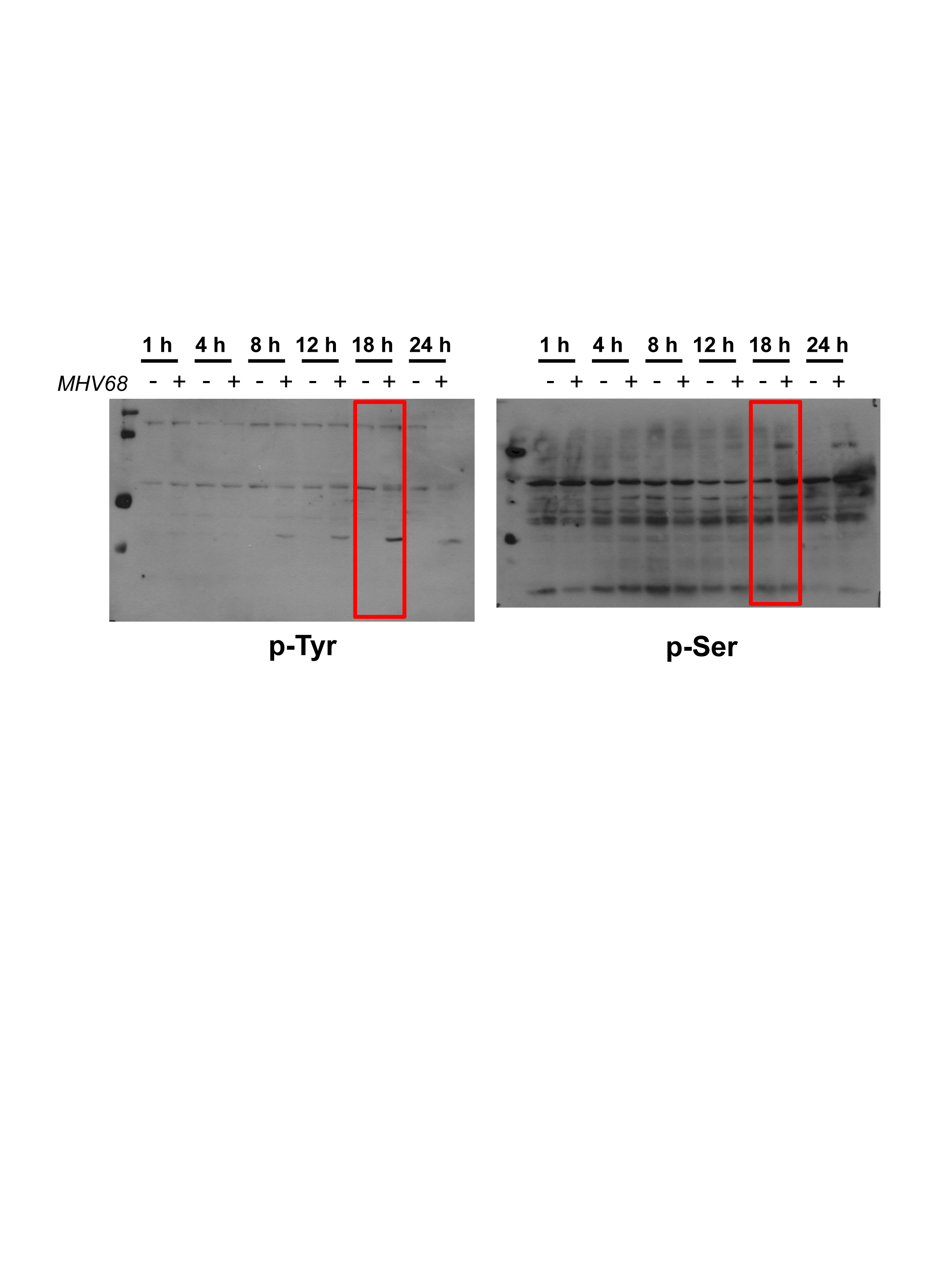

Supplement: Figure S1 — Identification of timepoints at which MHV68 elicits changes in phosphorylation patterns during productive infection. 3T3 fibroblasts were mock-infected or infected with MHV68 at MOI = 5 PFU/cell. Cells were harvested at the indicated times post-infection, and proteins were resolved by SDS-PAGE. Immunoblot analyses were performed to detect infection-related differences in proteins phosphorylated on Tyr or Ser. 18 h timepoints (red box) were selected for label-free quantitative phosphoproteomic analyses. (TIF) [file ppat.1003583.s001.tif]

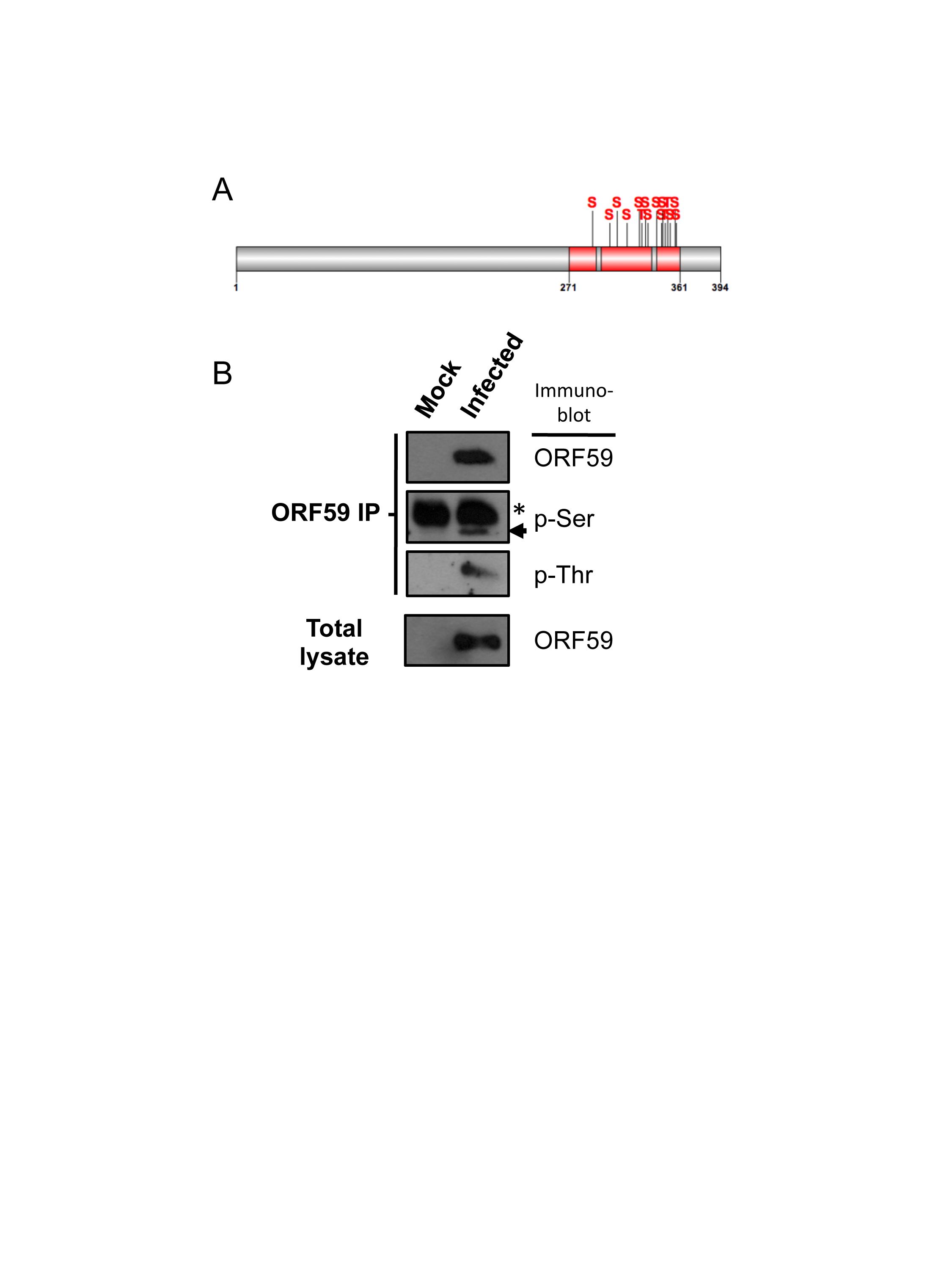

Supplement: Figure S2 — ORF59 is a viral phosphoprotein. (A) Schematic representation of MHV68 ORF59 highlighting sequenced amino acids and phosphorylated residues in red. (B) 3T3 fibroblasts were mock-infected or infected with MHV68 at MOI = 5 PFU/cell. Cells were harvested 18 h post-infection, and ORF59 was captured by immunoprecipitation. Immunoprecipitates and total cell lysates were resolved by SDS-PAGE and transferred to nitrocellulose. Immunoblot analyses were performed to detect ORF59, phospho-Ser, and phospho-Thr. The asterisk corresponds to IgY heavy chain. The arrowhead corresponds to precipitated ORF59. (TIF) [file ppat.1003583.s002.tif]

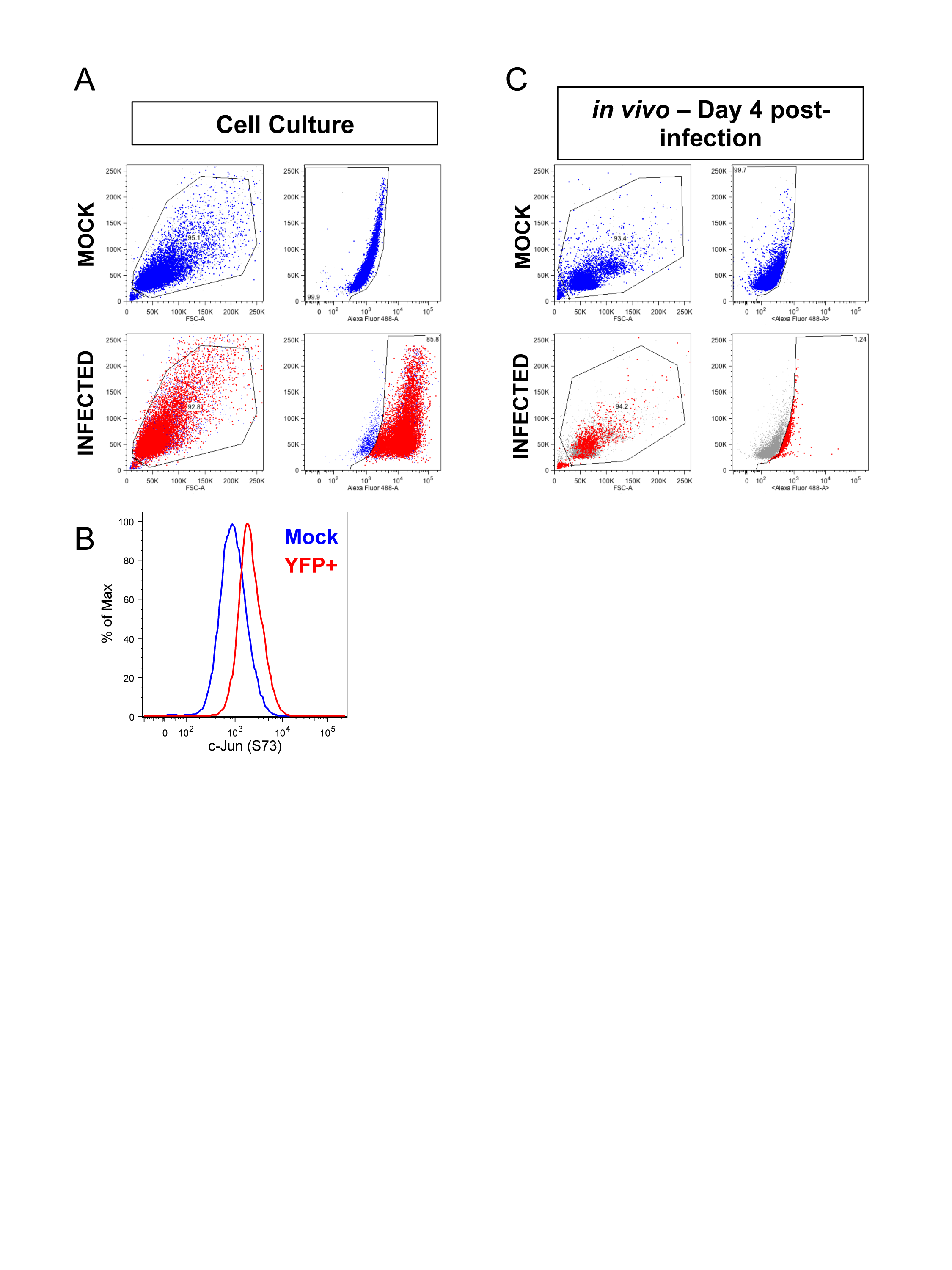

Supplement: Figure S3 — Enhanced c-Jun S73 phosphorylation occurs specifically in infected cells. (A and B) 3T3 fibroblasts were mock-infected or infected with H2B-YFP-expressing MHV68 (Collins and Speck, 2012) at MOI = 5 PFU/cell. 18 h post-infection cells were harvested and processed for intracellular antigen staining to detect H2B-YFP (A) or c-Jun phosophorylated on S73 (B) by flow cytometry. H2B-YFP expressing cells were identified by flow cytometry following staining with goat anti-GFP (Rockland Immunochemicals) and donkey anti-goat Alexa 488 (Invitrogen). H2B-YFP+ cells were identified relative to mock-infected cells. c-Jun (S73) intensities were determined in mock-infected and H2B-YFP+ cells by flow cytometry following staining with PE-conjugated rabbit anti-phospho-S73 c-Jun (Cell Signaling Technology). Isotype controls were used to determine background staining intensity (not shown). (C) 6–8 week-old C57BL/6 mice were intraperitoneally mock-inoculated or inoculated with 106 PFU of H2B-YFP-expressing MHV68. 4 days post-infection, mice were sacrificed, and splenocytes were isolated and processed as in A and B to detect H2B-YFP and S73-phosphorylated c-Jun (See Figure 3). Shown are representative dot plots to demonstrate the relative percentage of H2B-YFP+ cells detected in infected animals and the gating strategy used to segregate YFP− and YFP+ cells for comparative analyses of c-Jun phosphorylation within the same animal. A total of 3 mock-inoculated and 4 H2B-YFP MHV68 inoculated animals were used for these experiments. (TIF) [file ppat.1003583.s003.tif]

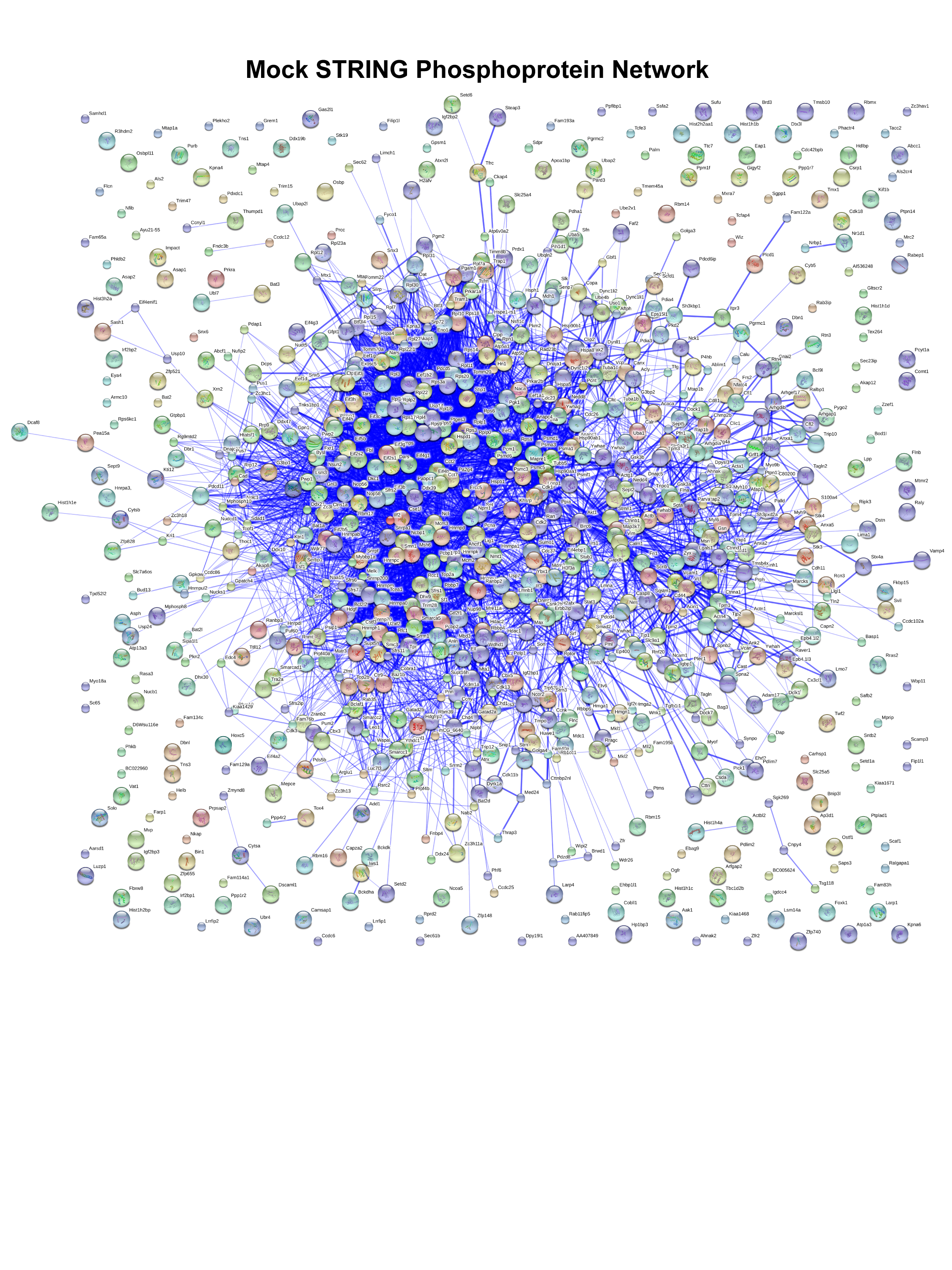

Supplement: Figure S4 — STRING analyses predict phosphoprotein interaction networks for uninfected control cells. Proteins identified in phosphoproteomic analyses were submitted to STRING (Szklarczyk et al., 2011) for bioinformatic predictions of phosphoprotein interaction networks. STRING-predicted total protein networks for mock-infected cells are shown. Networks are provided as confidence views where the edge weight (lines connecting nodes) correlates to confidence of the interactions. Interactions were predicted with standard STRING-defined confidence values of 0.400. STRING data where used to generate phosphoprotein networks presented in Figure 4. (TIF) [file ppat.1003583.s004.tif]

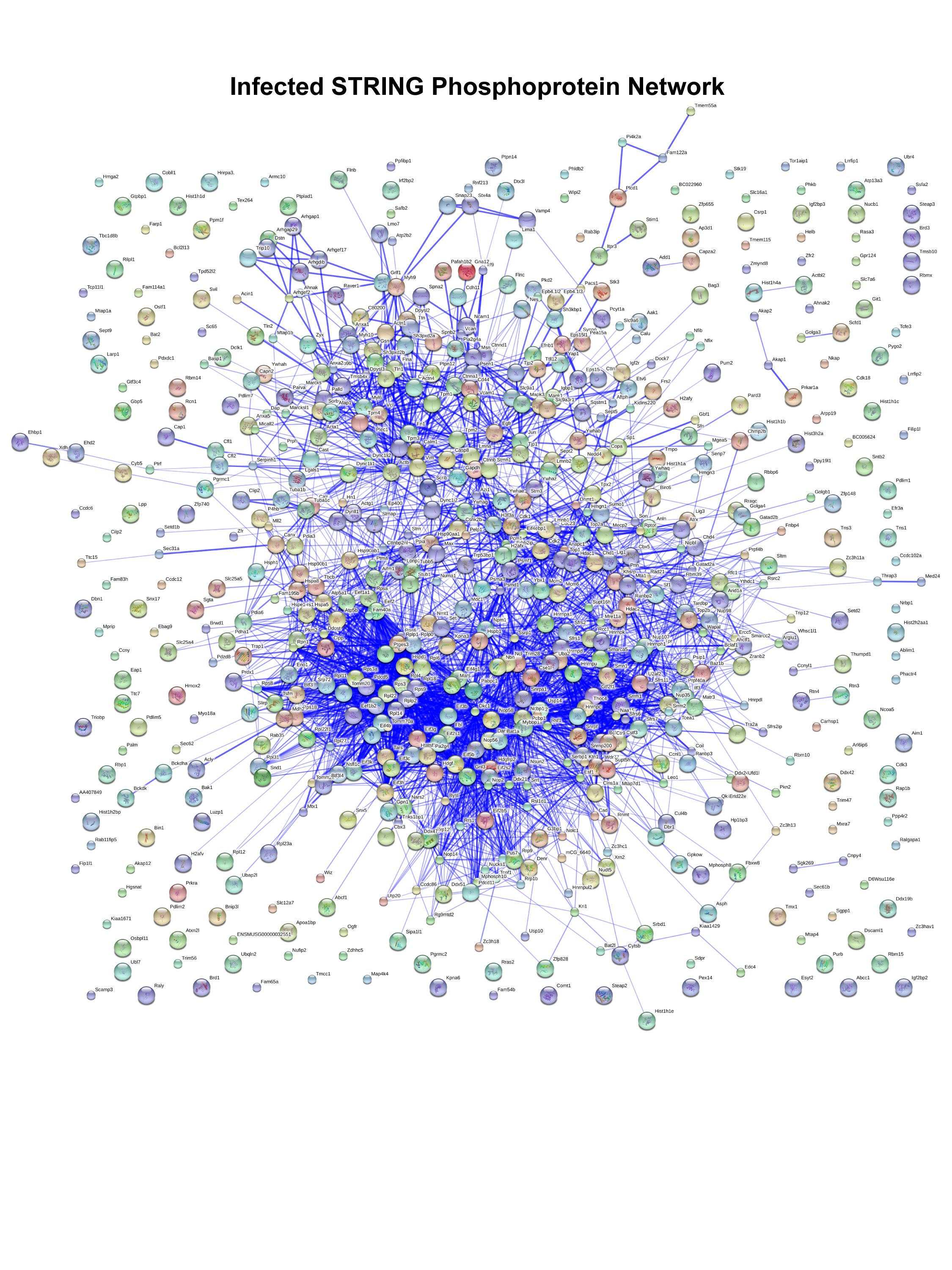

Supplement: Figure S5 — STRING analyses predict phosphoprotein interaction networks for MHV68-infected cells. Proteins identified in phosphoproteomic analyses were submitted to STRING (Szklarczyk et al., 2011) for bioinformatic predictions of phosphoprotein interaction networks. STRING-predicted total protein networks for infected cells are shown. Networks are provided as confidence views where the edge weight (lines connecting nodes) correlates to confidence of the interactions. Interactions were predicted with standard STRING-defined confidence values of 0.400. STRING data where used to generate phosphoprotein networks presented in Figure 5. (TIF) [file ppat.1003583.s005.tif]

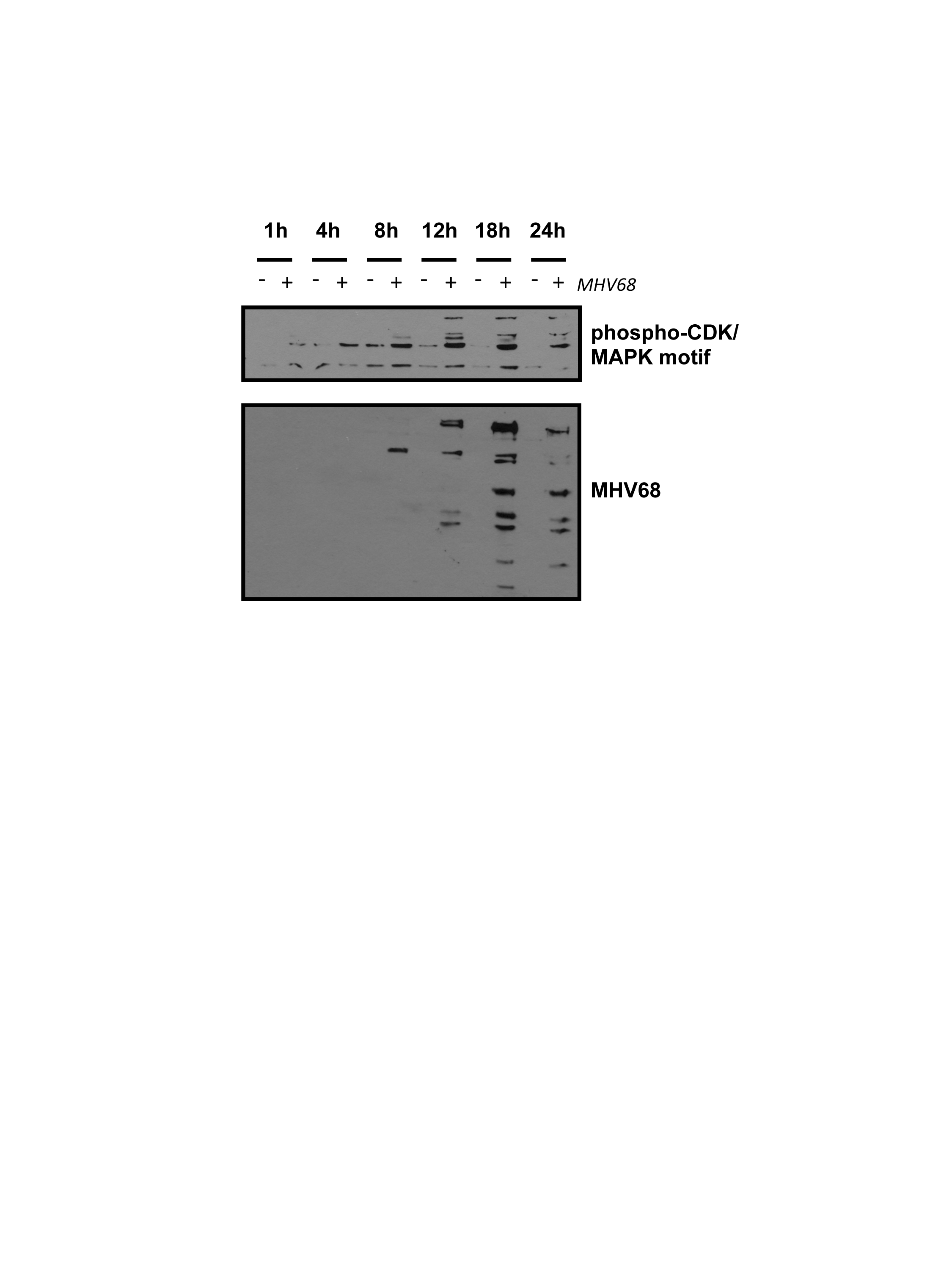

Supplement: Figure S6 — Identification of timepoints at which MHV68 elicits MAPK/CDK phosphorylation during productive infection. 3T3 fibroblasts were mock-infected or infected with MHV68 at MOI = 5 PFU/cell. Cells were harvested at the indicated times post-infection, and proteins were resolved by SDS-PAGE. Immunoblot analyses were performed to detect infection-related differences in proteins phosphorylated on CDK/MAPK motifs (antibody from Cell Signaling Technology). Blots were also probed with MHV68 antiserum to demonstrate progression through the lytic replication cycle. (TIF) [file ppat.1003583.s006.tif]
