# Supplementary material for: Phosphoproteomic Analyses Reveal Signaling Pathways That Facilitate Lytic Gammaherpesvirus Replication
Source: PLoS Pathog. 2013 Sep 19;9(9):e1003583. doi: 10.1371/journal.ppat.1003583 (PMC3777873; doi:10.1371/journal.ppat.1003583)
Supplement: Table S4 — Mass spectrometry technical information. (DOC) [file ppat.1003583.s010.doc]

Supplemental Table 4. Parameters used for protein quantification and identification.

| **Parameter** | **Value** |
| --- | --- |
| **Version** | 1.0.12.31 |
| **Released** | TRUE |
| **Peptide FDR** | 0.01 |
| **Max. peptide PEP** | 1 |
| **Protein FDR** | 0.01 |
| **Min. peptide Length** | 6 |
| **Min. unique peptides** | 1 |
| **Min. peptides** | 1 |
| **Reverse string** | fIPI |
| **Contaminant string** | CON_ |
| **Peptides used for protein quantitation** | All |
| **Min. ratio count** | 2 |
| **Number of threads** | 4 |
| **Re-quantify** | TRUE |
| **Keep low-scoring versions of identified peptides** | TRUE |
| **Fasta file** | C:\MaxQuant_1.0.12.31\MaxQuant\db\Con_ipi.MOUSE.v3.82_.fasta |
| **Experimental design file** | C:\MaxQuant_1.0.12.31\MaxQuant\raw\mouse_data\combined\experimentalDesignTemplate.txt |
| **Variable modifications** | Acetyl (Protein N-term);Oxidation (M);Phospho (ST);Phospho (Y) |
| **Fixed modifications** | Carbamidomethyl (C) |
| **Database** | Con_IPI_mouse_v3.82 |
| **MS/MS tol** | 0.5 Da |
| **Username** | edmondsonrickyd |
| **Max. msm file size [MB]** | 350 |
| **Top MS/MS peaks per 100 Da** | 6 |
| **SILAC type** | singlets |
